# Translating viral genetic data to PRRSV-2 cross-neutralization using machine learning

**DOI:** 10.3389/fimmu.2026.1875393

**Published:** 2026-07-10

**Authors:** Nakarin Pamornchainavakul, Jing Huang, Igor A. D. Paploski, Dennis N. Makau, Clarissa P. Ferreira, Venkatramana D. Krishna, Declan C. Schroeder, Maxim C.-J. Cheeran, Kimberly VanderWaal

**Affiliations:** 1 Department of Veterinary Population Medicine, College of Veterinary Medicine, University of Minnesota, St. Paul, MN, United States; 2 Department of Biomedical and Diagnostic Sciences, University of Tennessee, Knoxville, TN, United States

**Keywords:** AlphaFold, antigenicity, computational immunology, cross-neutralization, genetic diversity, immunological protection

## Abstract

**Background:**

Swine herd immunization through modified live vaccination or live virus inoculation is a core strategy for controlling porcine reproductive and respiratory syndrome (PRRS), one of the most economically important endemic diseases in the United States that is caused by PRRSV-2. While antigenically homologous PRRSV-2 viruses are expected to elicit better immunological cross-protection, questions remain as to how genetic similarity translates to the degree of cross-protection elicited between the immunizing strain and subsequent challenge virus. For PRRSV-2, virus neutralization (VN) assays are commonly used to approximate humoral immune responses and to assess cross-variant vaccine efficacy; however, these assays are labor-intensive and difficult to scale for rapid immunization planning.

**Methods:**

Here, we developed a machine learning–based predictive model for PRRSV-2 cross-neutralization using viral genetic sequence data to address this gap. Virus neutralization assays were performed across a panel of viral isolates and anti-sera, representing a variety of contemporary and vaccine-like PRRSV-2 variants. Our internal dataset comprised 219 cross-neutralization pairs involving nine viral isolates and 25 antisera generated from animals inoculated with six isolates (immunizing viruses). Neutralization titers were classified as above versus below average using mean standardized log_2_ titers as the cutoff. Candidate predictors included residue-wise amino acid property differences, overall viral genetic distances, and protein structural comparison metrics. Eleven machine learning algorithms were trained and evaluated using internal and external test sets.

**Results:**

The best models, which utilized predictors from protein ectodomain features, effectively estimated whether a virus-antisera pair would exhibit above versus below average neutralization, achieving balanced accuracies of 87–92% on internal data, and 71–89% across three external VN datasets. Key predictors included amino acid properties in GP5 decoy epitope and hypervariable regions, residues within GP2–GP3 epitopes, and overall genetic distance.

**Conclusion:**

These models are publicly available through a web-based tool (https://stemma.shinyapps.io/PRRSLoom-NeutralizationPredictor/) for end-users to estimate the neutralization potential between genetically distinct PRRSV-2 viruses. Our integration of machine learning, *in vitro* experimental data, and webtool bridges experimental research with real-world application in veterinary decision-making.

## Introduction

Porcine reproductive and respiratory syndrome (PRRS) is caused predominantly by PRRS virus type 2 (PRRSV-2) in the United States, with PRRSV-1 occurring at a very low prevalence (1, 2). The high incidence and severe clinical impacts of PRRSV-2 hinder swine industry productivity at multiple stages, from abortions in breeding herds to poor growth and respiratory distress in growing pigs, making PRRS one of the most significant burdens on U.S. livestock production (3–5). As of 2020, the disease is estimated to cost the industry over 1.2 billion dollars annually (6). Despite more than three decades of PRRS endemicity and the implementation of various control and prevention strategies, the incidence of the disease remains high. Herd immunization remains the most effective measure to mitigate clinical outcomes (7–9). Nearly half of PRRS-positive breeding herds use vaccines, with some also employing live virus inoculation (LVI) of a farm-specific wildtype virus (10, 11). However, studies and field observations suggest that immunization often results in variable cross-protection against heterologous infections amid the ongoing diversification of PRRSV-2 (12, 13).

PRRSV-2 is classified as *Betaarterivirus americense*, an enveloped positive-sense, single-stranded RNA virus belonging to the family *Arteriviridae* and the order *Nidovirales (*14). Its RNA genome contains seven open reading frames (ORFs). ORF1 is translated by the host ribosome into a protein complex responsible for viral transcription and replication. ORFs 2 to 7 are transcribed into a set of sub-genomic mRNAs, which are then translated into structural proteins (SP), including envelope glycoproteins (GPs; GP2, GP3, and GP4 of the minor glycoprotein complex encoded by ORF2a, ORF3, and ORF4, and GP5 and M of the major glycoprotein complex encoded by ORF5 and ORF6, respectively) and the nucleocapsid (N) protein encoded by ORF7 (15–17). Among these, ORF5 has been the most sequenced and studied gene because of its high genetic variability and its role in harboring a broadly neutralizing epitope (18, 19).

Classification of PRRSV-2 into lineages (genetic distance <11%), sub-lineages (<8.5%), and variants (~2.5%) based on ORF5 phylogenetic relationships has been widely implemented (20–25), largely because ORF5 sequencing is routinely used by veterinary practitioners to track epidemiological spread, support disease monitoring, and inform vaccine-related decision-making in field settings (25). Although exposure to genetically homologous viruses is generally considered to confer stronger immunological protection than to heterologous viruses (12, 26, 27), the relationship between ORF5 genetic distance and clinical cross-protection is variable and not well understood. Consequently, genetic similarity is used as an imperfect proxy for antigenic phenotype, complicating predictions of protective outcomes against outbreak variants. This uncertainty is particularly important for planning herd exposures through vaccination or controlled LVI, where a better understanding of the antigenic relationships among co-circulating PRRSV variants could help optimize immunization strategies and disease management practices aimed at minimizing production losses.

The purposes of immunization using modified live virus (MLV) vaccine or LVI are to induce both humoral and cell-mediated immune (CMI) responses against PRRSV, either type 2 or type 1 (*Betaarterivirus europensis*), the latter being predominant in European countries (28). Although CMI responses are generally delayed (29–31), they can target infection with heterologous PRRSV strains, though it has been shown that IFN-γ responses (a surrogate marker for T cell activation) vary in magnitude across viral isolates in a strain-specific manner (13, 32–34). Furthermore, neutralizing antibody titers from humoral responses are typically higher against homologous strains (12, 13, 32, 33). In other words, the humoral response tends to be more strain- or variant-specific. However, previous studies on PRRSV-1 have shown that homologous immunity can sometimes be less effective at reducing clinical signs and viremia than heterologous immunity (35), and that comparing the percent similarity of ORF5 sequences between vaccine and field viruses is not a reliable predictor of protection (36). Another study on PRRSV-2 reported that the level of heterologous neutralizing antibodies (within the same ORF5-based phylogenetic lineage – L1) was comparable to that of the L1 homologous strain, although with a different time to peak response, with both exceeding the titers observed for the lineage 5 homologous challenge (37). Collectively, these variations in humoral responses highlight that the relationship between immune responses and ORF5 sequence homology is complex and multifactorial, making it challenging to predict immune cross-reactivity solely through comparing the percent similarity between viruses based on ORF5.

Machine learning is a promising approach for predicting cross-neutralization properties of infectious diseases. Numerous studies have applied machine learning algorithms to predict whether a particular human immunodeficiency virus (HIV) strain is susceptible or resistant to therapeutic broadly neutralizing monoclonal antibodies (bNAbs) (38–41). Machine learning has also been used in swine influenza to predict antigenic distances from viral sequence data, illustrating its potential to estimate phenotypic differences and inform vaccine strain selection (42). For PRRSV-1, a previous study demonstrated that a machine learning algorithm could predict antigenic distances estimated from neutralization assays with 81% accuracy using viral glycoproteins (GPs) 2–5, and 75% accuracy using only GP5 amino acid sequences (43).

In the present study, we generated cross-neutralization titer data from a wide range of circulating PRRSV-2 variants in the United States. These data served as training data for developing machine learning algorithms to assess cross-neutralization and predict the potential for immune escape by emerging or newly introduced strains. We also integrated the best-performing predictive models into a web-based tool, which we publicly released to allow users to predict the neutralization level (high or low) of sera from animals immunized with a) major commercial MLVs against their uploaded PRRSV-2 ORF5 or complete structural gene sequences, or b) to estimate potential neutralization between any pair of uploaded viral sequences.

## Materials and methods

### Ethics statement

All animal and biosafety procedures in this study, including sample collection and handling, were reviewed and approved by the University of Minnesota Institutional Animal Care and Use Committee (IACUC; Protocol ID: 2210-40457A) and the Institutional Biosafety Committee (Protocol ID: 2201-39740H). The study was carried out in compliance with federal and state regulations, institutional guidelines, and the National Institutes of Health’s Guide for the Care and Use of Laboratory Animals.

### Virus propagation

Nine viral isolates representing contemporary and previously predominant PRRSV-2 variants, including MLV-like and LVI variants, were obtained from multiple collaborators and farms. The isolates (ORF5-based variant classification (25)) were IA/2014 (1A-unclassified) (44), NC174 (1A-unclassified) (37), JA1262 (1B-unclassified) (45), LVI-F4 (1C.2), NC134 (1C.3) (37), 46/2020 (1C.5) (46), LVI-F7/8 (1C.5), D11-052871 (5A.1) (47), and FL12 (8A.1) (48, 49) (**Supplementary Table 1**). Variant classification was performed with the March 2026 version of PRRSLoom-variants (25). PRRSV-2 isolates were propagated in either MARC-145 cells (RRID: CVCL_4540) or PAM-KNU cells (Applied Biological Materials Inc., Richmond, BC, Canada). Isolate 46/2020 was propagated in PAM-KNU cells, whereas all other isolates were propagated in MARC-145 cells. MARC-145 cells were maintained in minimum essential medium (MEM; Gibco™, Waltham, MA, USA) supplemented with 10% fetal bovine serum (FBS) and 10 mM HEPES. PAM-KNU cells were cultured in RPMI 1640 medium (Gibco™) supplemented with 10% FBS, 10 mM HEPES, 1 mM sodium pyruvate, and nonessential amino acids (Gibco™). During virus propagation, the FBS concentration in the culture medium was reduced to 2%. Virus stocks were collected after one freeze-thaw cycle and clarified by centrifugation at 400 × g for 5 min. To ensure consistent virus quantification across isolates, all virus stocks were titrated in MARC-145 cells and viral titers were determined as 50% tissue culture infectious doses (TCID_50_) using the Reed-Muench method (50).

### Experimental design for serum generation

To generate anti-sera against different PRRSV-2 isolates, sera were collected 60 days after *in vivo* virus inoculation. Inoculations were performed either at the University of Minnesota Veterinary Isolation Facility (VIF) or in commercial gilt development units (GDUs). At the VIF, 20 weaned pigs were sourced from a provider that meets the criteria for pathogen-free (specific pathogen free for PRRSV, Influenza virus, Mycoplasma, and other respiratory pathogens). Upon arrival, animals were randomly assigned to four groups (six animals in three groups and two animals in one group) and housed in separate isolation units. Blood (3–6 mL) and nasal swabs were collected from each pig to confirm the absence of PRRSV using RT-PCR with VetMAX PRRSV 3.0 Reagents (Thermo Fisher Scientific, USA). After three days of acclimatization, pigs were inoculated intramuscularly (1 mL in the neck muscle) and intranasally (1 mL total, 0.5 mL per nostril) with 1 × 10^5^ TCID_50_/mL of four different PRRSV-2 isolates, namely IA/2014 (variant 1A-unclassified) (46), JA1262 (variant 1B-unclassified) (45), 46/2020 (variant 1C.5) (46), or D11-052871 (variant 5A.1) (47), with one isolate administered to each group. At day 60 post-inoculation, when neutralizing antibodies should be at their peak levels (37, 51), we collected 50–110 mL of blood from all live animals at that time (n = 15) before euthanizing by barbiturate injection. Blood was centrifuged at 5000 × g for 15 min to obtain the sera. For the sera obtained from GDUs, two sets (n = 5 each) were generated at different GDU facilities from animals exposed to PRRSV-2 isolates LVI-F4 (variant 1C.2) and LVI-F9 (variant 1H.9) (**Supplementary Table 1**). These sera were collected from gilts acclimated in GDUs under controlled conditions. Inclusion criteria required that gilts had been exposed to only a single PRRSV isolate (LVI), the inoculum was available for sequencing, and serum could be collected at day 0, day 35, and day 60 post-inoculation. Consistent with the VIF protocol, sera collected at day 60 were used to capture peak neutralizing antibody titers.

### Virus neutralization

Neutralizing antibody responses were measured using a microplate-based focus reduction neutralization assay (52). Equal volumes of heat-inactivated serum and virus suspension containing a final inoculum equivalent to 500 PFU per well were mixed and incubated at 37 °C for 1 h. Virus concentrations were standardized from TCID_50_ titrations performed in MARC-145 cells using a standard TCID_50_-to-PFU conversion. Virus mixed with 1% PRRSV-negative porcine serum collected from PRRSV-naïve animals was included as the virus-only control, whereas maintenance medium alone served as the no-virus control. Following incubation, 100 µL of each serum–virus mixture was added to MARC-145 cell monolayers and incubated at 37 °C for 1 h. The inoculum was then removed and replaced with fresh maintenance medium (MEM supplemented with 2% FBS). At 11 h post-infection, cells were fixed with 4% paraformaldehyde and permeabilized with 0.2% Triton X-100. Infected cells were detected by immunostaining with a mouse monoclonal antibody against PRRSV-2 N protein (SR-30A; RTI, Brookings, SD, USA) diluted 1:10,000 and incubated for 1 h at 37 °C, followed by alkaline phosphatase-conjugated goat anti-mouse IgG (H+L) secondary antibody (Thermo Fisher Scientific) diluted 1:1,000 and incubated for 40 min at 37 °C. Color was developed using 1-Step™ NBT/BCIP Plus Suppressor Substrate Solution (Thermo Fisher Scientific) at room temperature for 20 min, followed by rinsing with water. Images of each well were acquired using an ImmunoSpot analyzer (CTL, Cleveland, OH, USA), and the numbers of foci were quantified using Fiji. The neutralization percentage for each well was calculated using the equation shown below. Neutralization percentages were plotted against serum dilution values, and a nonlinear regression curve was generated in GraphPad Prism 10 (Boston, MA, USA). The 50% neutralizing titer was interpolated from the fitted regression curve (**Supplementary Table 3**).


$$neutralization\;percentage=(1-\frac{Number\;of\;foci\;with\;Abs\;and\;viruses}{Number\;of\;foci\;with\;only\;viruses})\times 100\%$$

### RNA extraction, cDNA synthesis, library preparation, and sequencing

Viral genomes were sequenced from the inoculum corresponding to each isolate to confirm genome identity and to enable downstream comparative genomic and machine learning analyses. Total RNA was extracted from 200 µL of each viral isolate preparation using the NucleoMag^®^ Virus kit (TaKaRa Bio USA, San Jose, CA, USA) on a KingFisher™ Flex Magnetic Particle Processor (Thermo Fisher Scientific, Waltham, MA, USA), following the manufacturer’s instructions. First-strand cDNA synthesis was performed using a template-switching approach as previously described (53). Briefly, 4 µL of extracted RNA was combined with 1 µL of a 10 µM pooled primer mix and 1 µL of 10 mM dNTPs. The mixture was incubated at 70 °C for 5 min and immediately cooled to 4 °C. Subsequently, 2.5 µL of Template Switching Buffer, 0.5 µL of 75 µM template-switching oligonucleotide (TSO), and 1 µL of template-switching enzyme (New England Biolabs, Ipswich, MA, USA) were added. Reverse transcription was carried out at 42 °C for 90 min, followed by enzyme inactivation at 85 °C for 5 min. Second-strand cDNA synthesis was performed in a 25 µL reaction containing 2 µL of first-strand product, 0.25 µL of PrimeSTAR HS Polymerase (Takara Bio USA, San Jose, CA, USA), 5 µL of 5× PrimeSTAR buffer, 2 µL of 2.5 mM dNTPs, 1 µL of 10 µM TSO primer, and nuclease-free water to volume. PCR cycling conditions were 94 °C for 1 min; 30 cycles of 98 °C for 10 s, 60 °C for 15 s, and 68 °C for 2.5 min. Double-stranded cDNA was purified using CleanNGS magnetic beads (1:1 bead-to-sample ratio), washed twice with 80% ethanol, air-dried, and eluted in 20 µL of nuclease-free water. DNA concentration was quantified using the Qubit™ 1X dsDNA HS Assay Kit on a Qubit™ 4.0 Fluorometer (Thermo Fisher Scientific). Sequencing libraries were prepared using the Rapid Barcoding Kit (SQK-RBK114.24) (Oxford Nanopore Technologies, Oxford, UK) according to the manufacturer’s instructions. Barcoded library was pooled and loaded onto an R10.4 flow cell (FLO-MIN114) and sequenced on a GridION platform (Oxford Nanopore Technologies, UK) for 24 h using default run parameters. Genomes were reference-assembled against a selected set of PRRSV-2 genomes (GenBank accession numbers KP283400, MN073155, MN073102, MZ423533, MZ423535, and OL963970) using Minimap2 v2.24 (54) with the *map-ont* preset in Geneious Prime 2025.0.2. Consensus sequences were generated using a 60% agreement threshold and subsequently subjected to manual curation. Full-length consensus sequences were aligned using MAFFT v7.490 (55) with default parameters (200PAM/k = 2 scoring matrix; gap opening penalty: 1.53; offset value: 0.123).

### Outcome categorization and feature extraction

The mean virus neutralization (VN) titer for each serum–virus pair, consisting of 25 antisera from animals inoculated with six immunizing viruses tested against nine viral isolates, was log_2_-transformed because VN titers were determined using two-fold serial serum dilutions, with the endpoint defined as the highest dilution that inhibited viral replication in 50% of wells. This resulted in a 9 × 25 matrix, with each row corresponding to one of the nine tested isolates and each column corresponding to antisera from an animal exposed to a particular immunizing virus. Some viral isolates may be generally harder or easier to neutralize, potentially due to differences in receptor binding affinity or N-glycosylation, which create row-wise autocorrelation. Similarly, column-wise autocorrelation arises from animal-level effects (56–58). Thus, log_2_-titers were standardized across rows (general virus-level effects) and columns (animal-level effects) to ensure comparability across different serum–virus pairs. Briefly, data were standardized using successive z-score normalization steps (59). Values were first z-scored across rows (viruses) within each column (animal), followed by z-scoring across columns within each row. A standardized titer >0 indicates above-average neutralization between antiserum from immunizing virus A and isolate B, relative to the titers typically observed for antiserum from immunizing virus A against other tested isolates and for other antisera against isolate B. The standardized titers were further classified into binary categories using the mean of the transformed titers (z-score = 0) as the cutoff, defining low (≤ 0) and high (> 0) neutralization groups. This classification facilitated interpretation by distinguishing neutralizing titers that were higher or lower than average.

**Figure 1:**
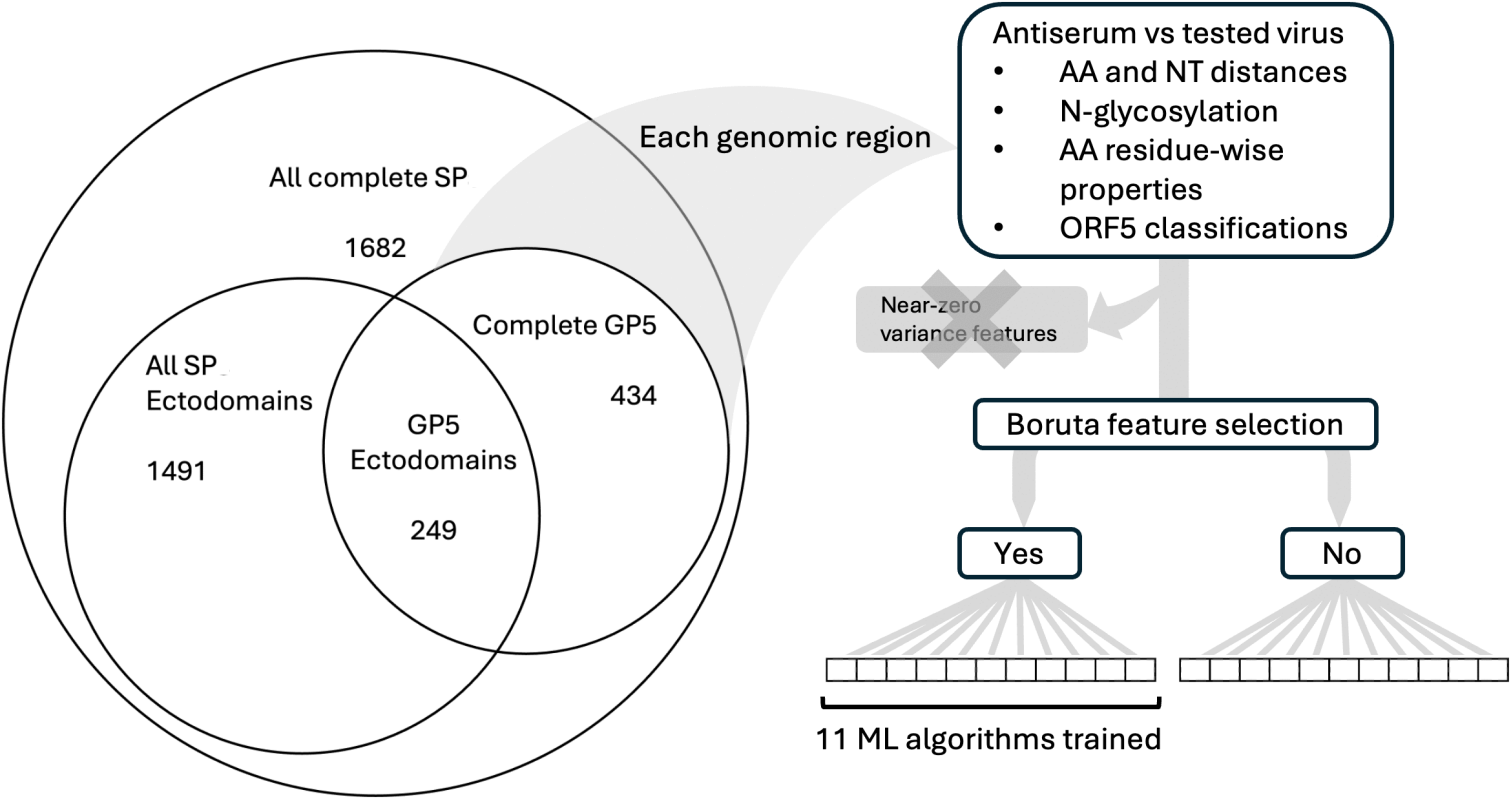
Development of machine learning models for PRRSV-2 neutralization prediction. Four feature sets were defined based on genomic coverage (all structural proteins [SP] vs. GP5) and protein regions (Complete vs. Ectodomains). The Euler diagram (left) illustrates the hierarchical relationship between these sets, where “Complete” regions encompass their respective “Ectodomain” subsets. The values shown represent the number of features unique to each nested layer, for instance, the total number of features for “Complete GP5” is the sum of its specific features (434) and the nested “GP5 Ectodomains” (249). For each region, features were generated by comparing immunizing and tested viruses, followed by removal of near-zero variance features and optional Boruta feature selection. Each feature set was then used to train 11 machine learning algorithms and evaluated using internal and external datasets.

Features used for training the different machine learning models in the subsequent step were primarily derived from comparative amino acid sequence metrics between the immunizing virus and the tested viral isolate. In order to test the contribution of different genomic regions in predicting the neutralizing response, we constructed four types of models that differed in which genomic regions were included in the feature sets. These included all complete structural proteins (SP), all SP ectodomains, complete GP5, and the GP5 ectodomains (**Figure 1**). For each model type, several features were computed, including overall nucleotide (nt_dist) and amino acid sequence divergence (aa_dist), measured using the “dist.dna” function in R’s ape v5.8.1 package (60) and Hamming distances (61), respectively; and differences in N-linked glycosylation patterns (gly or nogly) predicted from amino acid sequences containing N-X-S/T sequons (62); and residue-wise differences in the amino acid physicochemical properties.

Key amino acid physicochemical properties associated with viral protein conformation and applied previously in VN prediction for other viruses (41) were retrieved from the AAindex database via the bio3d v2.4.5 (63, 64) R package. These included the hydropathy index (65) (hydro), accessible surface area (66) (surf), average flexibility index (67) (flex), normalized frequency of β-turns (68) (btprop), isoelectric point (69) (pI), and net charge (70) (charge). These residue-wise differences were directional rather than absolute: for a given serum–virus pair (e.g., antiserum from immunizing virus A vs. isolate B), the feature value was calculated as the property value of the immunizing virus minus that of the tested isolate, so that reversing the pair (B vs. A) would yield an inverse value, unless the residues were identical. Residue-wise comparisons of amino acid identity (same or not) and, for non-identical residues, fundamental amino acid grouping (polar, nonpolar, acidic, basic) were also included as potential model features.

Additionally, we incorporated features describing the phylogenetic relationship between the immunizing virus and the tested viral isolate based on their ORF5 classifications from PRRSLoom-variants (25), including whether the pair belonged to the same variant (samevar), same sub-lineage (same_SL), or same lineage (same_L). As immunological protection against outbreak viruses is one of the insights animal health professionals typically infer from viral sequence homology (25), we also computed Spearman’s correlation coefficients (ρ) using the cor.test function in R (71) between each sequence-based nucleotide distance and antigenic similarity, where higher standardized VN titers indicate greater antigenic similarity, across all VN pairs.

To further capture potential differences in protein structure and their influence on measured neutralization, predicted protein structures for the minor and major glycoprotein complexes (GP2/GP3/GP4 and GP5/M, respectively) and the nucleocapsid protein (N) were generated using AlphaFold 2.0 (72). These structures were aligned and compared using the root mean square deviation (RMSD) or average atomic distance of superimposed predicted protein structures between each immunizing virus and tested viral isolate pair via the bio3d v2.4.5 (64) R package. In total, 3,856 features were generated for each of the 219 VN pairs and used for machine learning model training (**Figure 1**).

### Machine learning model training and test

For each of the four feature sets (all complete SP, all SP ectodomains, complete GP5, GP5 ectodomains), the dataset containing the outcome and all features was partitioned into training and test sets in an 80:20 ratio (179:40 pairs) using group-based stratified splitting. This approach ensured that all observations from the same immunizing virus–tested viral isolate pair remained in the same fold to prevent data leakage. All near-zero variance features were first removed using the caret v7.0.1 package (73). Two feature configurations were then created (1): the feature set without further selection, and (2) the feature set after Boruta feature selection via the Boruta v9.0 package (74). When applied, Boruta retained only features identified as important by comparison with randomized shadow features.

Eleven machine learning algorithms were compared for each feature configuration: random forest (RF) (75), gradient boosting machine (GBM) (76), elastic net (*glmnet*) (77), generalized linear model (GLM) (78), support vector machine (SVM) (79), linear discriminant analysis (LDA) (80), k-nearest neighbors (KNN) (81), decision tree (*rpart*) (82), naive Bayes (NB) (83), XGBoost (84), and light gradient boosting machine (LightGBM) (85). Most models were trained using 10-fold cross-validation via the caret v7.0.1 package (73), with the area under the receiver operating characteristic curve (ROC AUC) computed using the pROC v1.19.0.1 package (86) as the optimization metric. The GLM was trained using base R v4.5.1’s “*glm*” function (71) with the binomial family, while LightGBM was trained using the lightgbm v4.6 package (87) with binary logistic loss, a learning rate of 0.05, 100 boosting rounds, and early stopping after 10 rounds without validation improvement.

Model performance on the test set was evaluated using metrics derived from confusion matrix components (true positives: *TP*, true negatives: *TN*, false positives: *FP*, and false negatives: *FN*): sensitivity (*TP/[TP + FN], aka* recall), specificity (*TN/[TN + FP]*), positive predictive value (PPV) (*TP/[TP + FP]*, *aka* precision), negative predictive value or NPV (*TN/[TN + FN]*), accuracy (*[TP + TN]/[TP + TN + FP + FN]*), balanced accuracy (*[sensitivity + specificity]/2*), F1-score (*2 × precision × recall/[precision + recall]*), and ROC AUC. Results from all 22 combinations (11 algorithms × 2 configurations) for each of the four feature sets (88 total combinations) were ranked based on balanced accuracy.

### Model test on external data

We also evaluated our trained machine learning models on external data using three independent data sources that measured neutralizing antibody responses against diverse challenge viral isolates. The first dataset originated from Proctor et al. (2022) (13), in which pigs were vaccinated with the Prevacent^®^ PRRS MLV vaccine (variant 1D.2). Each group of six pigs was challenged 28 days post-vaccination with one of four viral isolates (VR2332 [variant 5A.1], NC174 [variant 1A-unclassified], NADC20 [variant 9E-unclassified], or NADC30 [1C-unclassified]). Serum samples collected 14 days post-challenge were tested for neutralizing antibody responses against the homologous challenge strain using a fluorescent focus neutralization (FFN) assay (13). Because sera were collected 42 days post-vaccination and 14 days post-challenge, the measured neutralizing antibody responses may reflect vaccine-primed memory immune responses rather than primary responses to the challenge viruses. Therefore, we hypothesized that challenge viruses that were more antigenically similar to the vaccine virus would elicit a stronger anamnestic neutralizing response than challenge viruses that were more antigenically distinct. We classified an observation as “high” for the model outcome categorization if the neutralization titer was ≥1:4, consistent with the positive cutoff defined in that study, and compared the observed and predicted high/low classifications using a confusion matrix.

The second external dataset was derived from Kim et al. (2013) (88), in which antisera against the prototype strain VR2332 (5A.1) were generated in 22 PRRSV-naïve pigs experimentally inoculated with 10³ TCID_50_/ml of VR2332. Sera were collected 2–3 months post-inoculation, and samples with VN titers ≥1:64 were pooled and standardized to a VN titer of 1:64 for subsequent assays. Neutralizing activity against 69 diverse field isolates (variants: 1A-unclassified, 1A.1, 1D-unclassified, 1F.1, 4-unclassified, 5A.1, 5B-unclassified, 8A.1, 8B-unclassified, 9A-unclassified, 9D-unclassified, 9E-unclassified, and undetermined) collected between 1999–2002 was evaluated using FFN assays. Among the 69 isolates, 10 exhibited a substantial reduction in replication (VN titer ≥1:16) and were classified as susceptible (S), whereas the remaining 59 isolates (VN titer ≤1:8) were classified as resistant to neutralization (R). However, based on the deduced amino acid sequence homology to VR2332 (using the 95% and 94.5% cutoffs) described in Table 1 of Kim et al. (88), only 67 isolates could be clearly classified as S or R. We therefore used this subset as an external benchmark to distinguish high (S) versus low (R) antigenicity for model evaluation.

The last external dataset was derived from Popescu et al. (2017) (89), in which VN assays were performed using sera from piglets experimentally infected with different PRRSV-2 isolates as part of studies conducted by the PRRS Host Genetics Consortium (PHGC) (90). Sera collected at 42 days post-infection were tested against a panel of homologous and heterologous viruses. For our evaluation, we extracted VN data from three groups of antisera generated against KS62 (8A.1), NVSL (8A.1), and KS06 (6.1), and their neutralizing activity against five viral isolates (KS62, NVSL, KS06, P129 [8C.1], and VR2332 [5A.1]). Because the number of animals contributing antisera differed across infection groups (nine for KS62, three for NVSL, and one for KS06; 13 antisera total), we applied successive standardization to the log_2_-transformed VN titers—first by viral isolate and then by animal—consistent with the procedure used for our internal dataset. We then calculated the median standardized log_2_ titer for each unique serum–virus pair (15 pairs total). Pairs with a standardized log_2_ titer >0 were classified as high, and those ≤0 as low, and these binary outcomes were used for model evaluation.

Our high/low neutralizing titer classification strategies were tailored to the experimental design of each external dataset. Successive standardization was applied only to the Popescu’s VN dataset (89) because it featured a cross-neutralization matrix with multiple immunizing and tested viruses, mirroring our training data. For the first two datasets, which tested only a single immunizing virus (13, 88), we utilized the cutoffs directly established in the original studies to prevent introducing artificial variance and account for inter-laboratory assay variability. This approach acknowledges that external “high” and “low” classifications may not reflect uniform biological neutralization levels, given that FFN/VN titers can be assay- and study-specific. For example, in Proctor et al. (2022), the comparison evaluates model-predicted higher versus lower neutralization potential against observed detectable versus non-detectable responses.

Because ORF5 sequences were the only consistently available data across all external datasets, feature extraction was restricted to the complete GP5 and GP5 ectodomain feature sets. Consequently, we evaluated 44 GP5-based models, using 24 immunizing–tested virus pairs from Proctor’s VN dataset, 67 pairs from Kim’s VN dataset, and 15 pairs from Popescu’s VN dataset. Finally, we selected two GP5 models that ranked among the top three in balanced accuracy on the internal test set and consistently achieved >70% balanced accuracy across all three external test sets, along with one SP model that demonstrated the highest balanced accuracy on the internal test set. For these models, feature contributions were assessed using SHAP (SHapley Additive exPlanations) values (91) via the *shapviz* v0.10.2 package (92), providing observation-level interpretability by estimating each feature’s contribution to individual predictions. Most visualizations were generated using the *ggplot2 (*93) package.

## Results

### Virus genome assembly and annotation

Genome sequences were obtained from the inoculum of each immunizing virus and tested viral isolate, including IA/2014 (GenBank accession: MZ423533), 46/2020 (MZ423535), JA1262 (PZ332180), FL12 (PZ332179), NC134 (PZ332184), NC174 (PZ332185), LVI-F7/8 (PZ332182), D11-052871 (PZ380673), LVI-F4 (PZ332181), and LVI-F9 (PZ332183). Genome assemblies generated from 1,076–42,980 reads per sample yielded genome lengths of 14,913–15,413 nt. Pairwise nucleotide identity across complete genomes ranged from 82–99.5%, reflecting a genetic diversity that spans from closely related to highly divergent isolates. Amino acid identity was 85–99.5% for GP5 and 78–100% for other structural proteins. Glycoproteins (GP2–GP4) showed comparable variability (78–99%), while the M and nucleocapsid (N) proteins were more highly conserved (86–100% and 91–100%, respectively) (**Supplementary Figure 1**, **Supplementary Table 2**).

### Virus neutralization cannot be fully determined by overall viral genetic relationship

**Figure 2:**
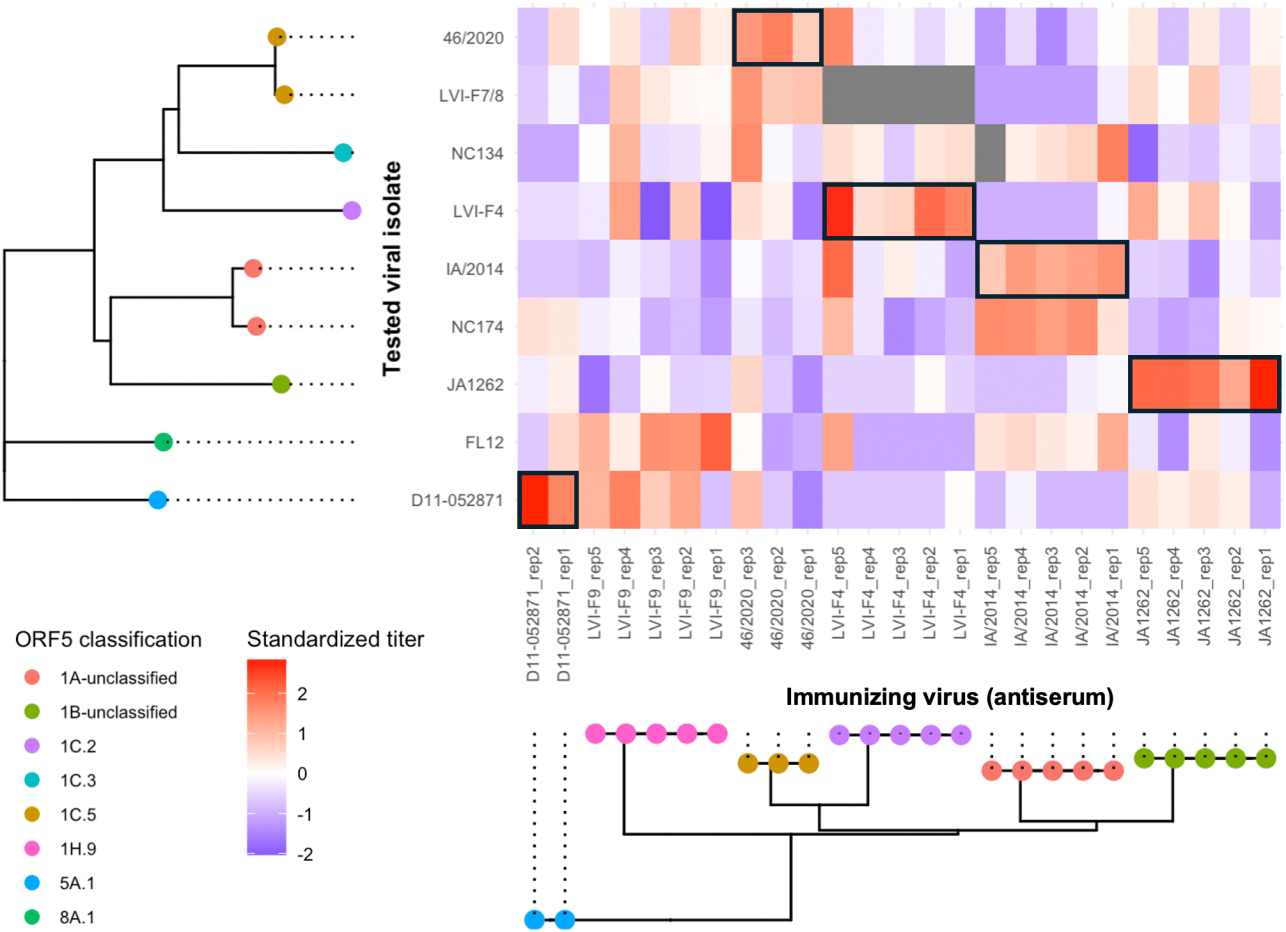
Standardized cross-neutralization titers. The heatmap displays cross-neutralization titers between nine tested viral isolates (rows; left phylogeny) and antisera against six different immunizing viruses (columns; bottom phylogeny). Titers were log_2_-transformed and sequentially standardized by tested virus and by animal. Red tones indicate values above the mean (z-score > 0; classified as high titer), whereas blue tones indicate values at or below the mean (z-score ≤ 0; classified as low titer). Grey cells represent missing data. Black borders in the heatmap indicate 100% homologous pairs where the immunizing virus was identical to the tested viral isolate. Tip colors in both phylogenetic trees denote PRRSV-2 variants based on ORF5 classification.

Nine viral isolates were cross-neutralized using sera obtained from six groups of animals, each inoculated with different viral isolates. Because there were multiple animals per group, this yielded a total of 219 neutralization titer observations (**Supplementary Table 3**). The tested isolates and immunizing viruses were classified into eight variants, representing vaccine-like, previously predominant, and currently dominant variants circulating in the U.S. (**Supplementary Table 1**). Neutralization titers for each serum–virus (VN) pair were measured by the microplate-based focus reduction neutralization assay, converted to a log_2_ scale. The resulting matrix was standardized across both rows (tested viruses) and columns (antisera derived from different animals) to minimize confounding from virus and animal variation. A standardized titer of zero was used as the cutoff to classify outcomes as high or low, where “high” indicates above-average neutralization (**Figure 2**). Median (IQR) raw neutralization titers were 67.0 (31.6–180.3) and 12.7 (<8–28.8) for the high- and low-neutralization categories, respectively.

**Figure 3:**
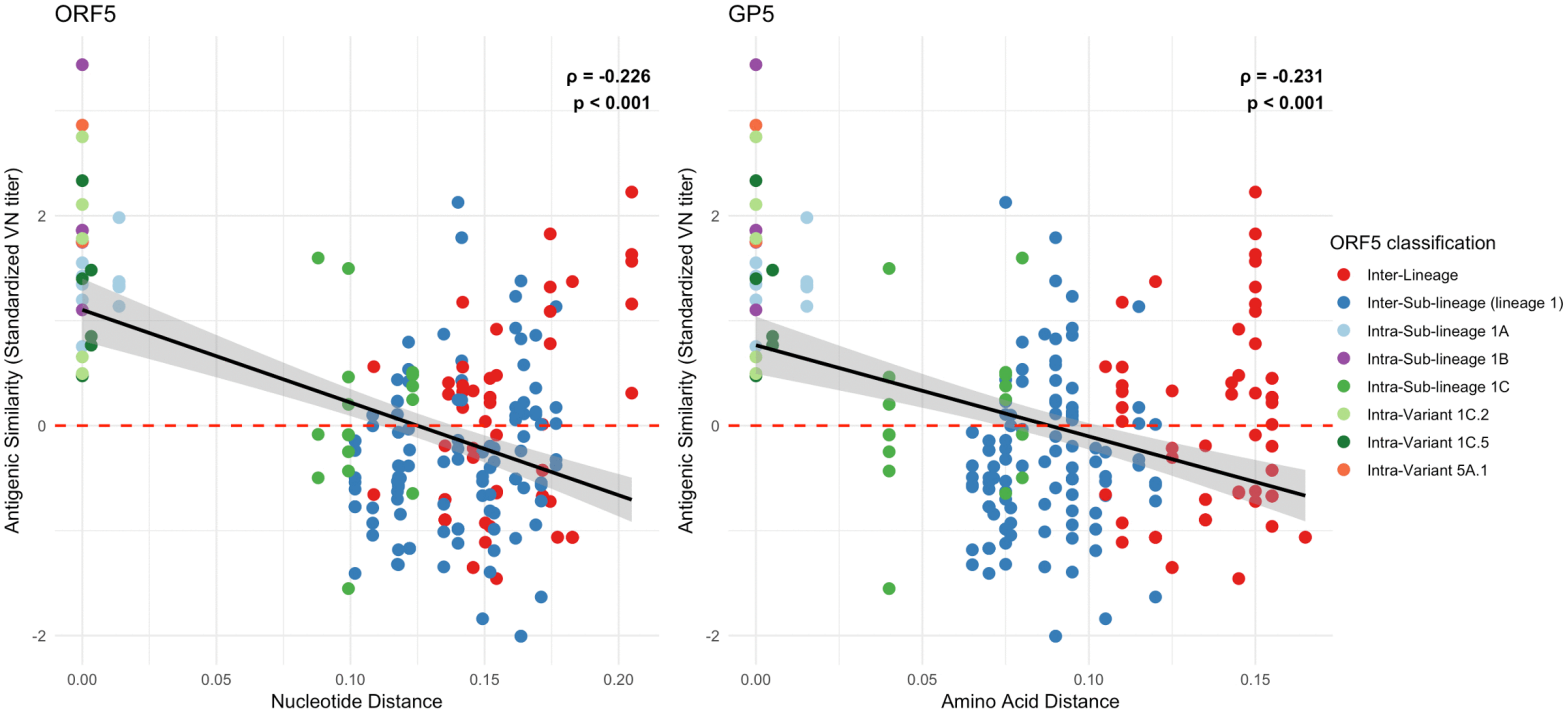
Correlation between antigenic and genetic similarity. Scatter plots show the relationship between standardized virus neutralization (VN) titers and genetic distance, measured as nucleotide distance in ORF5 (left) and amino acid distance in GP5 (right). The horizontal red dashed line denotes the mean VN titer (z-score = 0). Black lines indicate fitted linear regression models, with shaded bands representing 95% confidence intervals. Summary statistics for each association, including Spearman’s correlation coefficient (ρ) and p-value, are shown in the upper right. Point colors indicate the relationship between immunizing–tested virus pairs based on ORF5 classification, spanning variant- to lineage-level groupings.

Although genetic homology, particularly based on the ORF5 gene, is often used as indicator of potential antigenic homology, only modest correlations were observed. Across all six structural genes, antigenic similarity (standardized titer) was negatively correlated with both nucleotide and amino acid distances for all genes (p < 0.01), with Spearman’s ρ ranging from -0.36 (M protein) to -0.19 (ORF4). These correlations were primarily driven by VN pairs with <2% nucleotide and amino acid distances, which consistently showed above average neutralization titers. Such pairs were typically within the same variant and sub-lineage based on ORF5 classification. However, high antigenic similarity was consistently observed only among pairs within the same variant (often identical viral isolates), and not necessarily between pairs within the same-sub-lineage. Pairs within the same sub-lineage, particularly sub-lineage 1C, the most genetically diverse group, did not always exhibit high antigenic similarity. Likewise, VN pairs from different ORF5-based sub-lineages or lineages (with nucleotide distance >8% on ORF5 and >2.5–5% across other structural genes) displayed wide variation in antigenic similarity, suggesting that antigenic relationships cannot be reliably inferred solely from genetic distance once viruses exceed sub-lineage-level divergence in ORF5 or ~2.5% divergence across other structural genes (**Figure 3**; **Supplementary Figures 2**-**6**). In ORF5, for example, viruses with genetic distances ranging from 8 – 20% formed a cloud around the “average” line (standardized titer = 0), where variations in genetic distance did not translate to predictable, linear changes in antigenic similarity (**Figure 3**).

### Ectodomain models yield higher accuracy than the full-length protein and show comparable accuracy between GP5- and SP-based models

We evaluated multiple machine learning algorithms and feature sets to identify models capable of accurately predicting high/low virus neutralization outcomes. Two GP5 ectodomain models demonstrated consistently high performance across all internal and external evaluations: K-nearest neighbor (KNN) and light gradient boosting machine (LightGBM) (**Supplementary Table 5**).

The KNN model using 36 Boruta-selected GP5 ectodomain features achieved the highest balanced accuracy (0.92) on the internal test set (n = 40). It showed perfect positive predictive value (1.00) and specificity (1.00), with a sensitivity of 0.83, resulting in an F1-score of 0.91 and a ROC AUC of 0.88. The LightGBM model using the full set of 171 GP5 ectodomain features (without Boruta selection) performed comparably, with a balanced accuracy of 0.87 and a ROC AUC of 0.93 on the internal test set (**Table 1**, **Supplementary Table 4**).

**Table 1:** Predictive performance of selected top-performing models on internal and external test sets (13, 88, 89).

Feature set	Algorithm	Number of features	Test set	Balanced accuracy	Accuracy	Sensitivity	Specificity	PPV	NPV	F1-score	ROC AUC
GP5 ectodomains	KNN	36	Internal	0.92	0.93	0.83	1.00	1.00	0.88	0.91	0.88
			(13)	0.71	0.67	0.43	1.00	1.00	0.56	0.60	0.54
			(88)	0.89	0.90	0.87	0.90	0.72	0.96	0.79	0.82
			(89)	0.72	0.73	0.67	0.78	0.67	0.78	0.67	0.82
	LightGBM	171	Internal	0.87	0.88	0.83	0.91	0.88	0.87	0.86	0.93
			(13)	0.80	0.83	1.00	0.60	0.78	1.00	0.88	0.93
			(88)	0.76	0.63	1.00	0.52	0.38	1.00	0.55	1.00
			(89)	0.75	0.73	0.83	0.67	0.63	0.86	0.71	0.78
SP ectodomains	LightGBM	1165	Internal	0.90	0.90	0.89	0.91	0.89	0.91	0.89	0.96

Among GP5-based models, both the KNN and LightGBM models demonstrated consistent generalization across three external VN datasets representing distinct viral diversity. The Proctor et al. (13) dataset (n = 24) reflected neutralization data from contemporary viruses, including lineage 1, 5, and 9 viruses tested against serum derived from a lineage 1 vaccine. On this dataset, LightGBM achieved the highest balanced accuracy (0.80) with perfect sensitivity (1.00), while KNN reached a balanced accuracy of 0.71, showing perfect specificity (1.00) but lower sensitivity (0.43). The Kim et al. (88) dataset (n = 67) represented predominantly pre-2010 isolates (lineages 1, 4, 5, 8, and 9) evaluated against serum raised to the lineage 5 PRRSV-2 prototype. On this dataset, KNN achieved a balanced accuracy of 0.89 with high sensitivity (0.87) and specificity (0.90), whereas LightGBM achieved a balanced accuracy of 0.76, characterized by perfect sensitivity (1.00) but lower specificity (0.52). The Popescu et al. (89) dataset (n = 15) captures non-lineage 1 neutralization profiles, comprising lineage 8 and 6 viruses tested against sera from lineages 5, 8, and 6. On this dataset, both models performed comparably, with balanced accuracies of 0.72 (KNN) and 0.75 (LightGBM). Overall, both KNN and LightGBM generalized robustly across temporally and genetically diverse external VN datasets, albeit with complementary sensitivity–specificity tradeoffs suggesting different operating characteristics depending on application context (**Table 1**, **Supplementary Table 4**).

For models incorporating all structural proteins (SP), LightGBM applied to SP ectodomains (without Boruta feature selection; 1,168 features) achieved the highest performance on the internal test set (balanced accuracy = 0.92; precision = 1.00; specificity = 1.00; ROC AUC = 0.92). This model could not be evaluated on the external dataset because whole-genome sequences were unavailable. When predicted structure-comparison features (RMSD) were removed (1,165 features remained), LightGBM maintained comparable performance (balanced accuracy = 0.90) and showed an improved ROC AUC (0.96) (**Table 1**, **Supplementary Table 6**), indicating that RMSD contributed minimally to predictive accuracy while adding complexity and computational cost to protein structure prediction.

Taken together, the KNN and LightGBM models were the best-performing GP5-based approaches, while the LightGBM model trained on SP ectodomains without RMSD features was the optimal SP-based predictor. Across both GP5 and SP feature sets, models trained on ectodomains outperformed those using full-length protein sequences, indicating that ectodomain-only features provided greater predictive power for neutralization outcomes (**Supplementary Tables 4**, **6**).

### Features that contributed most to predictions were derived from GP5 hypervariable regions and GP2–GP3 epitopes

Features contributing most to the predictions of the selected models were identified using SHAP (SHapley Additive exPlanations) values (91). The GP5-based LightGBM model, which performed slightly worse than KNN on the internal test set (balanced accuracy = 0.87) but achieved consistent accuracy across all external test sets (balanced accuracy = 0.75–0.80), used all 171 ectodomain-focused features without additional selection via Boruta.

**Figure 4:**
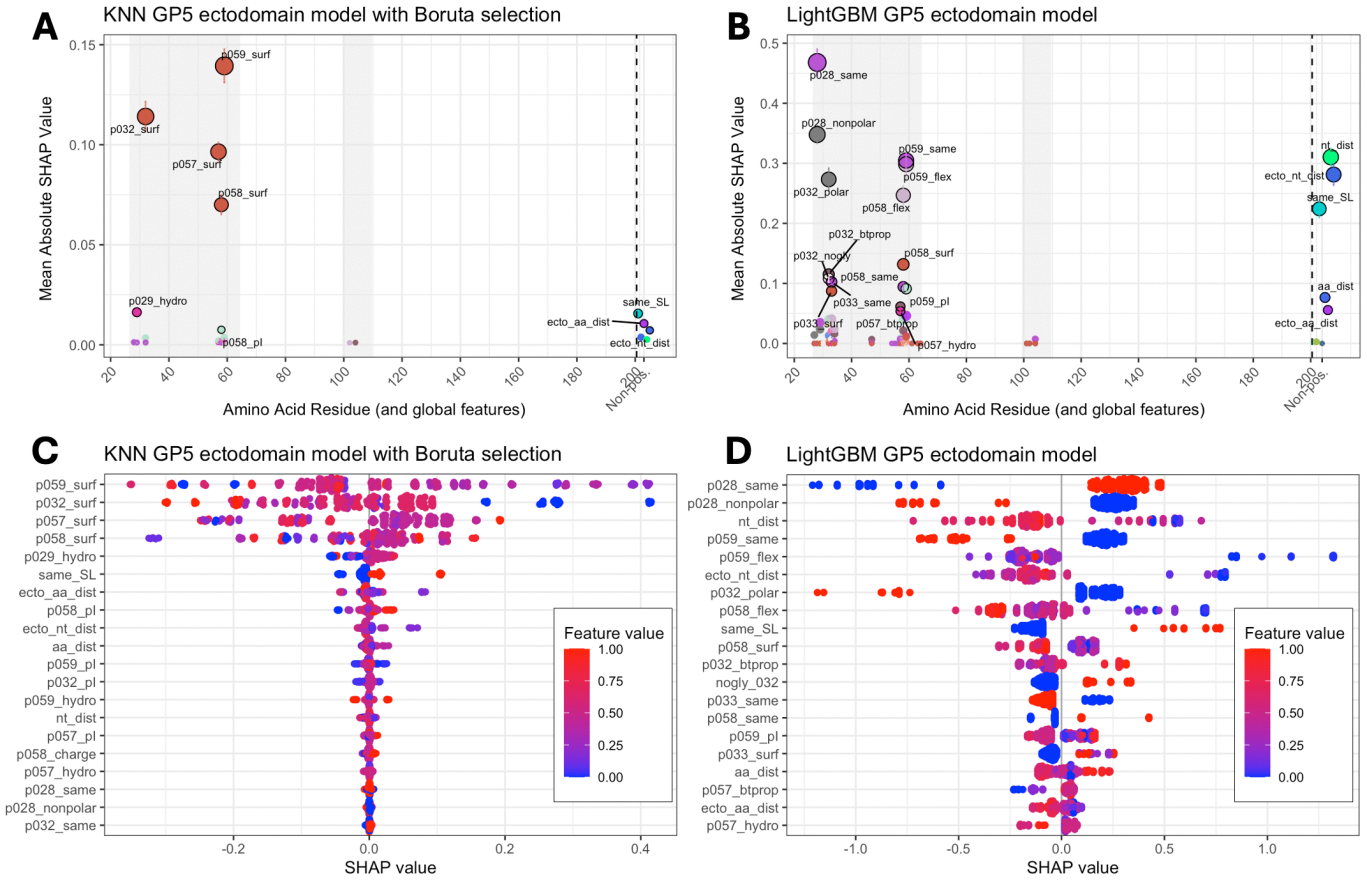
Key features of the best-performing GP5 ectodomain models. **(A)** (KNN) and **(B)** (LightGBM) show feature importance across GP5 residues using mean absolute SHAP values, with larger values indicating greater influence on predictions. Point size and position reflect SHAP magnitude, and colors denote residue-wise feature types. The grey region marks the GP5 ectodomain, while global (non–residue-specific) features appear to the right of the dashed line. **(C)** (KNN) and **(D)** (LightGBM) show SHAP beeswarm plots, where each point represents an observation colored by feature value (red = high, blue = low); positive and negative SHAP values indicate contributions toward higher and lower predicted neutralization titers, respectively. Full definitions of feature abbreviations are provided in the Methods section.

The top 20 SHAP-ranked features included both global indices such as nucleotide identity, sub-lineage co-membership, and residue-wise metrics. Key residue positions included residues 28, 32, 57, 58, and 59. The model incorporated multiple amino acid properties, including identity, polarity group, average flexibility index, accessible surface area, and normalized β-turn frequency. The effects of global indices (e.g., ORF5-wide or ectodomain nucleotide distances and sub-lineage labels) were comparable in magnitude to residue-wise features (**Figure 4B**).

Clear patterns were observed between feature values and the directionality of effects quantified by SHAP values, with many binary features showing distinct SHAP separations. For example, identical residue 28 was associated with positive SHAP values (increasing the likelihood of a high neutralization prediction), whereas non-identical non-polar residue 28 in both viruses was associated with negative SHAP values (lowering that likelihood). Similar trends were observed for continuous features such as nucleotide distances, flexibility index, and β-turn frequency (**Figure 4D**).

The GP5-based KNN model, which performed best on the internal test set (balanced accuracy = 0.92) and showed variable performance across the three external test sets (balanced accuracy = 0.71–0.89), used a smaller feature set comprising 36 ectodomain-focused features selected by Boruta. These included several overall indices and residue-wise amino acid comparison metrics derived from eight amino acid residues (28, 29, 32, 57, 58, 59, 102, and 104). Most of these residues overlapped with those identified as important in the LightGBM model, although KNN relied on fewer amino acid properties overall. Among these, differences in the accessible surface area at residues 32 and 57 to 59 ranked among the top 20 SHAP ranks and showed the strongest contributions on predictions (**Figure 4A**). Compared with the LightGBM model, relationships between feature values and SHAP values were less distinct in the KNN model, although a few patterns were observed. For instance, small differences in accessible surface area at residue 32 were associated with higher antigenic similarity, while similar hydropathy values at residue 29 tended to produce lower antigenic similarity (i.e., negative SHAP values) (**Figure 4C**).

Despite differences in feature composition and SHAP patterns, features that were consistently ranked by both models as important for predicting neutralization were in functionally and antigenically relevant regions of GP5 (**Table 2**). These included residues located within the GP5 non-neutralizing decoy epitope (residues 27–31), an immunodominant region that diverts the immune response toward non-neutralizing antibodies and delays the development of broad neutralizing antibody responses (18). This region is retained when the signal peptide cleavage occurs between residues 26 and 27 but absent when cleavage occurs between residues 31 and 32 (18, 94). Other important residues were located in hypervariable region 1 (residues 32–36), where glycosylation can shield the adjacent broad neutralizing epitope B (21, 95–98), and hypervariable region 2 (residues 57–61), which contains a glycosylation site at residue 57–59 and corresponds to epitope C, known to mediate homologous neutralization and restrict epitope B accessibility (19, 88, 89, 97, 99, 100).

**Table 2:** Amino acid residues contributing to the top 20 features of each best-performing model.

Gene	Residue	Best models			Importance					Source
		GP5 KNN	GP5 lightGBM	SP lightGBM	Positively selected	Potential N-glycosylated	Hyper-variable	Epitope	Findings	
GP2	56			X					–	–
	88			X					–	–
	102			X					–	–
	121			X	X			X	Immunodominant linear B-cell epitope.	(104, 108, 109)
	182			X	X				–	(104)
	188			X	X				–	(104)
	198			X				X	G198V mutation decreases neutralizing antibody titers.	(107)
GP3	79			X					–	–
	96			X					P96S mutation is associated with resistance to broadly neutralizing antibodies.	(89)
	134			X					–	–
	152			X		X			Essential for proper protein folding; loss of this glycan (together with residues 29 and 160) reduces viral production.	(105, 106)
GP5	28	X	X					X	Decoy epitope that delays the induction of broadly neutralizing antibodies.	(18, 94)
	29	X		X	X			X	Decoy epitope; the A29S mutation blocks signal peptide cleavage site 1 (26|27).	(18, 22, 94, 102)
	32	X	X	X	X	X	X		Signal peptide cleavage site 2 (31|32); the N32S mutation alters signal peptide processing and creates an alternative glycosylation motif, while N32K is found in neutralization-resistant haplotypes.	(18, 22, 94, 98, 99, 101–103)
	33		X		X	X	X		N33S mutation removes a potential glycosylation site (change in immune evasion)	(18, 22, 94, 99, 102, 103)
	57	X	X			X	X	X	Epitope C; a glycosylation site specific to sub-lineage 1A. The N57D mutation enables escape from homologous neutralization, and K57E is found in neutralization-resistant haplotypes.	(19, 89, 97, 99, 101)
	58	X	X		X	X	X	X	Epitope C; N58Q/E/K mutations eliminate glycosylation and have been identified in highly pathogenic Chinese strains.	(19, 22, 88, 97, 99, 100, 102–104)
	59	X	X		X	X	X	X	Epitope C; an infrequent glycosylation site in sub-lineages 1C/D, with a unique 59R residue identified in NADC30-like strains in China.	(19, 22, 97, 99, 102, 103, 111)

Together, these residue groups flank the conserved broad neutralizing epitope B of PRRSV-2, and likely contribute to immune evasion by reducing effective neutralization (89, 101). These regions are also frequently found to be under positive selection (22, 99, 102–104), and rank amongst the most influential features for accurate neutralizing titer class prediction.

Both global and residue-wise features were identified as major contributors in the SP-based LightGBM model. Several of these sites have been reported in relation to immunogenicity, whereas others have not been previously associated in viral phenotype or antibody interactions. The most influential residue-level features were predominantly located in GP2 and GP3, with a few in GP5 that overlapped with important residues highlighted by the GP5-based models, although with lower contribution in the SP-based framework.

Top residue-wise contributors ranked by absolute SHAP values included GP3 residue 96 (identity and normalized β-turn frequency), consistent with a previous findings linking mutations at this site with resistance to broadly neutralizing antibodies (89). Important features also included N-linked glycosylation at GP3 residue 152, one of several conserved GP3 glycans essential for proper protein folding, where loss of this glycan—together with those at residues 29 and 160—reduces infectious viral production (105, 106).

**Figure 5:**
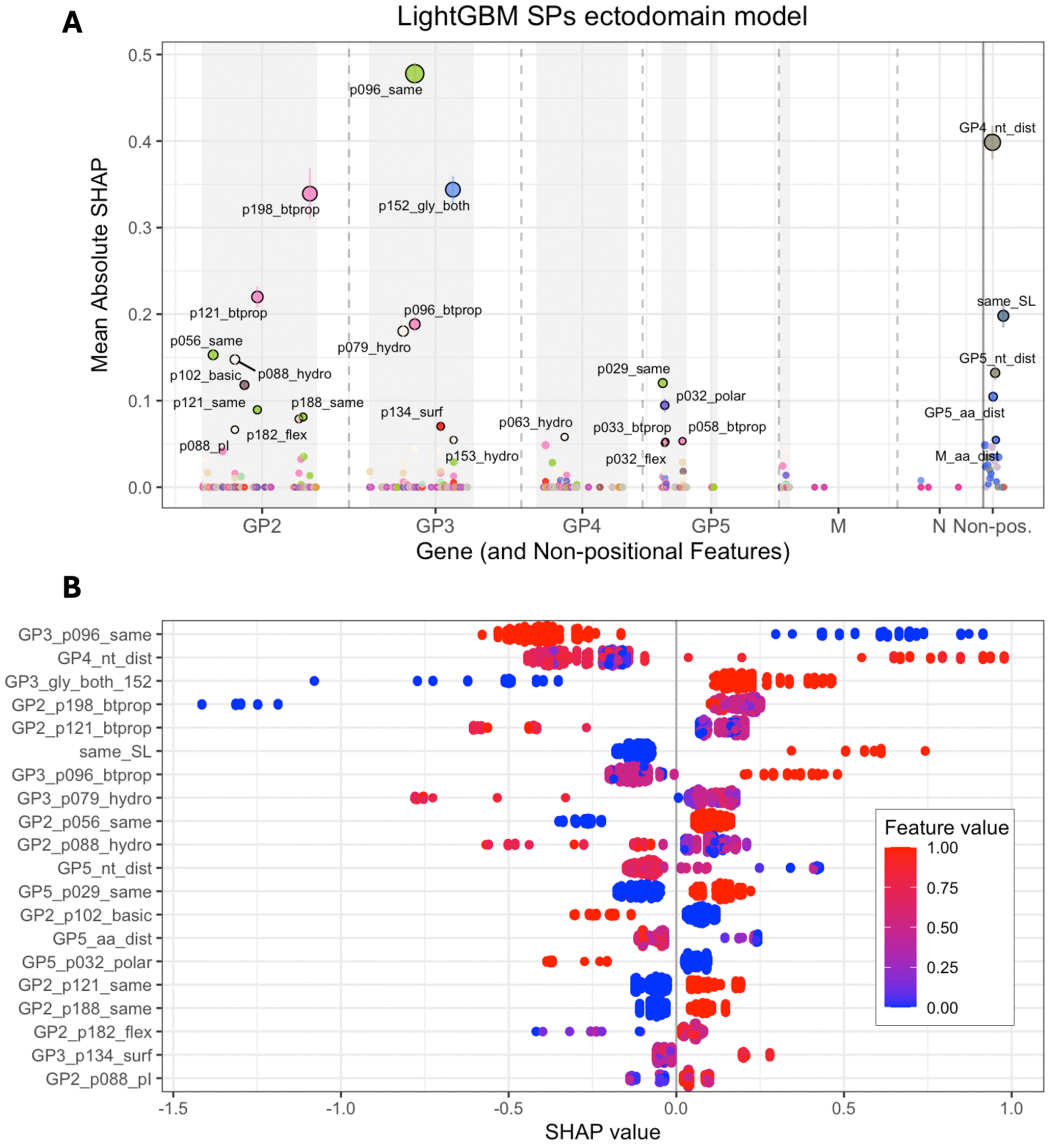
Key features of the best-performing structural protein ectodomain LightGBM model. **(A)** Feature importance across all structural protein residues (GP2 to N) based on mean absolute SHAP values, where larger values indicate greater influence on model predictions. Point size and position reflect SHAP magnitude, and colors denote residue-wise feature types. Grey regions mark the ectodomains of each structural protein, while global (non–residue-specific) features appear to the right of the dashed line. **(B)** SHAP beeswarm plots, in which each point represents an observation colored by feature value (red = high, blue = low); positive and negative SHAP values indicate contributions toward higher and lower predicted neutralization titers, respectively. Full definitions of feature abbreviations are provided in the Methods section.

Additional contributors were the β-turn of GP2 residue 198, a site shown by reverse genetics to alter neutralization titers and proposed to function as an antibody-binding site (107), and both the β-turn and identity of GP2 residue 121, which lies within one of two linear neutralizing epitopes (108, 109) and has been identified as a positively selected site (104). Other influential features corresponded to known regions of functional or evolutionary importance. These included properties of positively selected residues in GP2 [e.g., residues 182 and 188 (104)], hypervariable regions in GP4 [e.g., residue 63 (110)] and GP5 [e.g., residues 32, 33, and 58 (19, 97)], and the GP5 decoy epitope [residue 29 (18, 94)] (**Figure 5A**, **Table 2**). Like the GP5-based LightGBM model, the association between each residue-wise feature and the SHAP value was generally interpretable. For instance, differences in amino acid identity at GP3 residue 96, shared glycosylation at GP3 residue 152, and small differences in the β-turn frequency of GP2 residues 198 and 121 were associated with positive SHAP values (**Figure 5B**).

For the global features, the GP4 nucleotide distance emerged as one of the strongest contributors in the SP-based model, followed by the ORF5 sub-lineage classification and the nucleotide and amino acid distances of GP5 (**Figure 5A**). These predictors showed a similar pattern of association with the SHAP values, i.e., neutralizing titers tended to be higher than average when the genetic distances between the immunizing–tested virus pairs were low. An exception was GP4, where several high-distance outliers were associated with positive SHAP values (**Figure 5B**).

It is also worth noting that these global features contributing strongly to the SP-based model were significantly correlated with other global predictors. For example, all genetic distance metrics within the minor structural proteins (GP2–GP4) showed Spearman’s ρ generally greater than 0.5. Thus, it is unlikely that GP4 diversity alone determines neutralization; rather, the model is likely using GP4 distance as a representative feature within a set of correlated predictors (**Supplementary Figure 8**). When features derived from predicted SP structural comparisons were removed from the model (to reduce computational demands), other global features increased in importance. Unlike the genetic-distance-based global features, these structure-based metrics were not strongly correlated with their corresponding gene-level distance measures (ρ generally < 0.5), except for the RMSD of the GP5–M heterodimer, which showed modest correlations with GP5 ectodomain nucleotide and amino acid distances (maximum ρ = 0.52–0.55) (**Supplementary Figures 7**, **8**).

### Machine learning-based PRRSV-2 neutralization predictor

**Figure 6:**
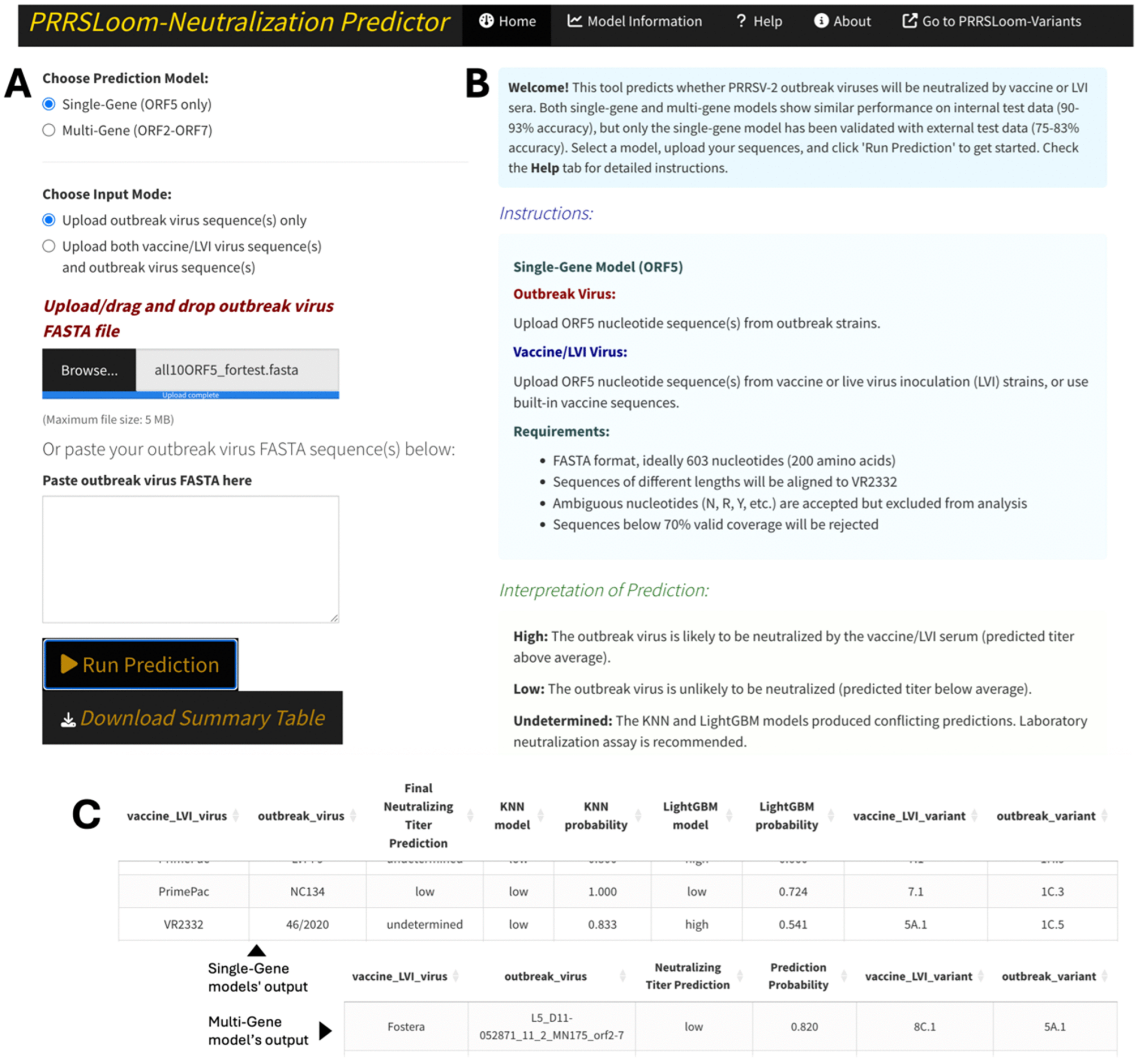
User interface of the PRRSLoom-Neutralization Predictor. **(A)** Webtool panel for selecting model type (GP5 or SP), comparison mode (built-in list of vaccines or user-supplied inputs), and input method (upload or paste). **(B)** Detailed instructions and guidance for interpreting predictive outcomes. **(C)** Summary table displaying predicted outcomes for all VN pairs.

To support practical use of the VN prediction framework, we developed the PRRSLoom–Virus Neutralization Predictor (https://stemma.shinyapps.io/PRRSLoom-NeutralizationPredictor), an interactive web-based tool built using R Shiny v1.11.1 (111). This application allows researchers and practitioners to obtain cross-neutralization predictions without requiring computational expertise or local software installation. It is designed for common field scenarios in which PRRSV-2 vaccine or LVI strains used on a farm correspond to the immunizing viruses, and the outbreak-causing virus represents the tested isolate in VN assays. The web interface provides two modes of operation. Users may upload viral sequence(s) and compare it against five pre-loaded vaccine strains (Fostera^®^ [GenBank accession: AF494042.1], Ingelvac ATP^®^ [DQ988080.1], Ingelvac MLV^®^ [AF066183.4], PrimePac^®^ [DQ779791.1], and Prevacent^®^ [KU131568.1]) and the PRRSV-2 prototype strain VR2332 (AY150564.1), or they may supply both custom immunizing viruses (e.g., vaccine/LVI or a virus that a herd has previous exposure towards) and challenge/outbreak viral sequences. Users can select either the single-gene (GP5) model or the multi-gene (SP) model (**Figure 6A**). Uploaded sequences are automatically aligned to the VR2332 reference. After input validation, the application executes the feature extraction pipeline, imputes missing features (especially those arising from non-translatable codons) using median values from our whole internal dataset (219 VN pairs). To maintain transparency, the tool reports which residues were imputed, allowing users to assess prediction reliability and determine if missing data might impact the classification. Finally, the processed features are passed through the best-performing models. Each model produces binary predictions (high or low neutralization) with associated probabilities. For the GP5 model, the final output employs a consensus approach: if the KNN and LightGBM models agree, the prediction is reported directly as either “high” or “low”; if they disagree, the result is labeled “undetermined.” Exportable outputs indicate the likely neutralization between each pair of sequences as predicted to be above-average titers (“high”), lower-than-average neutralization potential (“low”), or uncertain due to model disagreement (“undetermined”). For the SP model, a single prediction from the LightGBM model is reported (**Figure 6C**).

## Discussion

Virus neutralization (VN) assays are used to evaluate immunization efficiency and vaccine efficacy against PRRSV-2 isolates, but they are time-consuming, labor-intensive, and cannot be performed for every genetic variant detected across swine populations. Here, we translate VN benchwork into machine-learning models that approximate neutralization potential and immune escape risk for emerging or newly introduced PRRSV-2 strains. These models are intended to support assessment of whether viruses are likely to evade existing herd immunity elicited by vaccination, prior infection, or live virus inoculation (LVI), informing decisions related to herd intermixing among populations with different immune histories, LVI updates, and strain selection for autogenous vaccine development. Using a diverse PRRSV cross-neutralization dataset, we evaluated multiple algorithms and feature sets capturing amino acid and genetic differences between immunizing–tested virus pairs that are theoretically relevant to immunogenicity and immune response. High-performing GP5-based models, with balanced accuracies of 0.87–0.92, highlighted the importance of ectodomain amino acids in hypervariable regions together with overall genetic distance. In contrast, a structural protein–based model with a balanced accuracy of 0.90 identified informative features distributed across minor glycoprotein ectodomains and selected gene distances. These models are made publicly accessible through an R Shiny web tool, enabling practical reproducibility and reuse by the broader research and veterinary communities.

One of the primary motivations for sequencing PRRSV outbreak strains is to gain immunological insight that supports assessment of existing immune protection and evaluation of the likelihood that newly introduced viruses may evade herd immunity offered by host antibodies, typically inferred from genetic relatedness to previously encountered strains (25). This approach is grounded in numerous studies showing that antisera generated against viruses genetically homologous to a tested or challenge viral isolate are more likely to neutralize or protect against that virus than heterologous antisera-virus pairs (12, 13, 32, 33, 88). Genetic homology is typically defined based on ORF5 sequence similarity and is often interpreted as a proxy for antigenic similarity. Empirical data from our study partially support this concept, as homologous virus pairs with less than 2% genetic distance across structural genes (or belonging to the same ORF5-based variant) consistently exhibited high antigenic similarity. However, heterologous pairs displayed a wide range of antigenic similarity, in some cases exceeding that of certain homologous pairs (**Figure 3**).

While genetic homology provides an overall measure of viral similarity, it fails to capture the inherently directional nature of functional immunity. For example, in our study, reversing the roles of immunizing and tested viruses between the 46/2020 (1C.5) and LVI-F4 (1C.2) isolates resulted in conflicting neutralization classifications, highlighting the phenomenon of asymmetric cross-neutralization (i.e., immunity from past exposure to virus A neutralizes virus B, but not vice versa). This phenomenon has been reported in other viral systems (112, 113), and its underlying causes include but are not limited to, differences in epitope accessibility shaped by immunodominance hierarchies (114, 115) and glycan shielding (116, 117), antigenic variation that alters key neutralizing determinants between strains (118), and immune imprinting effects (original antigenic sin) (119, 120) that bias serum responses toward one virus’s antigenic landscape over another. All these factors influence the ability of antibodies elicited by virus A to neutralize virus B, but not vice versa. Motivated by this rationale, we extended feature generation in our predictive model beyond previous PRRSV-1 work, which relied primarily on binary residue-wise amino acid identity features (43). Instead, we incorporated residue-wise amino acid property differences between immunizing and tested viruses, adding directionality and improved immunological relevance.

Residues in PRRSV-2 GP5 hypervariable regions and the decoy epitope, as well as key sites in GP2 and GP3, emerged as major predictors of neutralization phenotype. Many of these sites lie within or near known immunogenic epitopes and glycosylation motifs that modulate antibody recognition. For example, glycan shielding, which can limit epitope accessibility, was identified at several top-ranked residues, and predicted glycosylation pattern comparisons at these positions were included in the best-performing models (21, 96, 117). Structurally, residues with high accessible surface area, along with β-turn propensity, flexibility, and polarity (top predictors in both the GP5 and SP models) are more likely to form conformationally exposed epitopes capable of interacting with B-cell receptors and circulating antibodies (121). However, some feature–SHAP associations were counterintuitive. For instance, neutralization titer tended to be higher when GP3 residue 96 differed between immunizing and challenge virus. This may result from several factors, such as pairs with similar amino acids at this residue potentially hindering neutralization, while certain differing residues may favor it, or multigenic effects where local residue properties interact with the broader protein context, or simply spurious, coincidental associations captured by the model. These variations in top features suggest that not only specific positions but also their structural and functional properties contribute to shaping PRRSV-2 neutralization sensitivity and immunogenicity. The removal of overall protein structure comparison indices (RMSD) from the SP model did not reduce predictive accuracy, likely because residue-wise and global features captured similar or more specific key information. Features that did not rank highly should not be considered unimportant. Rather, the models prioritized predictors that maximized performance given feature redundancy and correlations, and this may also reflect an incomplete understanding of the epitopes recognized by the host.

The lack of non-GP5 structural protein sequences in the external dataset prevented comprehensive validation of the SP model. Nevertheless, evaluation on the same internal test set showed that the SP model achieved higher ROC AUC and sensitivity than the GP5-based models, suggesting that incorporating additional structural protein information may capture predictive signals beyond GP5 alone. Supporting this interpretation, SHAP analysis identified influential residues in GP2 and GP3, including sites previously implicated in PRRSV neutralization (**Table 2**). Furthermore, GP2–GP4 encode minor envelope glycoproteins that contain neutralizing epitopes and glycosylation sites capable of mediating glycan shielding, both of which can influence antibody recognition and virus neutralization (89, 96, 106, 109, 122–125). Together, these findings suggest that GP2–GP4 features could provide biologically relevant information beyond GP5. However, the generalizability of this added value remains uncertain and requires independent validation. Future studies incorporating broader cross-neutralization datasets with complete structural protein or whole-genome sequences will be needed to validate and further refine the SP model.

Model selection was a critical step in this study given the wide range of machine learning algorithms and feature sets evaluated, particularly for the GP5-based models, which were tested against three independent external datasets. Our primary objective was to identify models that generalized robustly across both internal and external test sets, a criterion ultimately met by the KNN and LightGBM models. Although both models leveraged features derived from the same GP5 hypervariable regions, they differed substantially in feature complexity, interpretability, and performance stability across datasets. The KNN model achieved the highest balanced accuracy on the internal test set but exhibited more variable performance across external VN datasets. This pattern is consistent with the local, distance-based nature of KNN, which can be sensitive to covariate shift between training and external data distributions (126). In addition, its decision boundaries are inherently less interpretable, and SHAP analyses revealed less stable feature-attribution patterns. In contrast, the LightGBM model demonstrated more consistent external generalization and clearer, more stable SHAP relationships, as expected for tree-based gradient boosting models with exact TreeSHAP explanations (85, 127). Its robustness across heterogeneous datasets suggests improved handling of nonlinear feature interactions and distributional variability. This versatility may also explain why LightGBM outperformed other algorithms when features were expanded across all structural genes.

Prior biological knowledge has been adopted to improve machine learning model performance by mitigating noise from irrelevant features (128–131). We applied two complementary strategies to reduce number of features: Boruta feature selection, representing an automated reduction method without prior biological knowledge, and restriction to ectodomain residues, a biologically informed approach. Boruta, a supervised random forest–based method, aggressively reduced the feature set to as little as 3% of the original features, whereas the number of ectodomain features varied by gene (e.g., ORF7 lacks an ectodomain because it encodes the nucleocapsid protein). Across multiple machine-learning algorithms, ectodomain-restricted features generally outperformed the full feature set, independent of Boruta selection. Although Boruta contributed to some high-accuracy GP5 ectodomain models in internal testing, its performance was less reliable in external GP5 validation and internal SP analyses. This likely reflects its supervised nature, as it leverages outcome labels during training (74) and can overfit by selecting features that are highly predictive for the internal dataset but lack external generalizability. These results highlight that restricting the feature set to biologically grounded priors, such as antibody accessibility and known ectodomains, enhances model robustness and generalizability compared with purely data-driven feature reduction.

The development of PRRSV-2 neutralization prediction models in this study was primarily constrained by the limited availability of training and external validation data, as antiserum generation and virus isolation for standard VN assays require costly, controlled experiments to generate standardized, single-exposure sera. The technical difficulty of growing diverse PRRSV-2 isolates in the large volumes required for these assays often makes the generation of large-scale datasets logistically and financially challenging. Particularly, the external validation datasets were generated using different VN protocols, including differences in virus inoculum, assay formats, serum collection time points, endpoint definitions, and criteria used to classify neutralization outcomes. These methodological differences may introduce assay-dependent variation unrelated to true antigenic relationships and could influence estimates of model generalizability across studies.

Despite efforts to include diverse contemporary and vaccine-like variants, gaps remain, particularly the underrepresentation of VN pairs with ORF5 nucleotide distances between 2% and 8%, which may exhibit distinct feature or neutralization patterns. If future variants predominantly fall within this range, predictive performance may be reduced, necessitating ongoing model retraining and reevaluation as viral diversity evolves. The models were developed exclusively using PRRSV-2 viruses and antisera representative of diverse lineages circulating in the U.S., including variant 5A used in MLV vaccine. Therefore, their applicability to PRRSV-1 or highly divergent PRRSV-2 populations from geographic regions not represented in the training data remains unknown. Although the models performed well on external datasets, these U.S.-based datasets broadly overlapped the genetic diversity represented in the training data (median pairwise ORF5 genetic distance between internal and external datasets: 13.6–15.6% vs. 14.3% within the internal dataset). Future studies incorporating a broader range of genetically and antigenically diverse isolates could help better define the limits of genotype-based neutralization prediction and expand the model’s scope of application. Moreover, these models provide only indirect estimates of clinical protection. Neutralization reflects humoral immunity but represents just one of several factors influencing disease outcomes, alongside innate and cell-mediated immune responses, co- or secondary infections, and host variation. Accordingly, model outputs should be interpreted in combination with other immunological and epidemiological factors rather than as standalone predictors of protection.

## Conclusion

Neutralization across a broad diversity of PRRSV-2 viruses has been investigated to better understand the immunological complexity underlying its predictive value for protection against disease. In this study, we generated a diverse cross-neutralization dataset encompassing major contemporary, vaccine, and live virus inoculation (LVI) strains, and developed a publicly accessible resource for predicting neutralization potential using high-performing machine-learning models trained on these data. These predictive tools support animal health professionals in immunologically informed decision-making, including evaluating immune escape risk posed by circulating or introduced strains, evaluating compatibility among herds with distinct immune backgrounds, and informing adjustments to herd immunization strategies. Beyond these practical applications, future work can build on the model-identified amino acid features to advance mechanistic understanding of PRRSV-2 antigenicity, enhance vaccine design, and improve predictive frameworks for immune response and disease protection in more complex immunological contexts.

## Data Availability

The datasets presented in this study are deposited in the NCBI GenBank database under accession numbers MZ423533, MZ423535, PZ332180, PZ332179, PZ332184, PZ332185, PZ332182, PZ380673, PZ332181, and PZ332183, and the analysis code and supporting files are deposited in the GitHub repository at https://github.com/NakarinP/PRRSLoom-Neutralization-Predictor.git.
