# Supplementary material for: Translating viral genetic data to PRRSV-2 cross-neutralization using machine learning
Source: Front Immunol. 2026 Jul 10;17:1875393. doi: 10.3389/fimmu.2026.1875393 (PMC13395864; doi:10.3389/fimmu.2026.1875393)
Supplement: Supplementary file 2 [file Table2.docx]

**Supplementary figures**


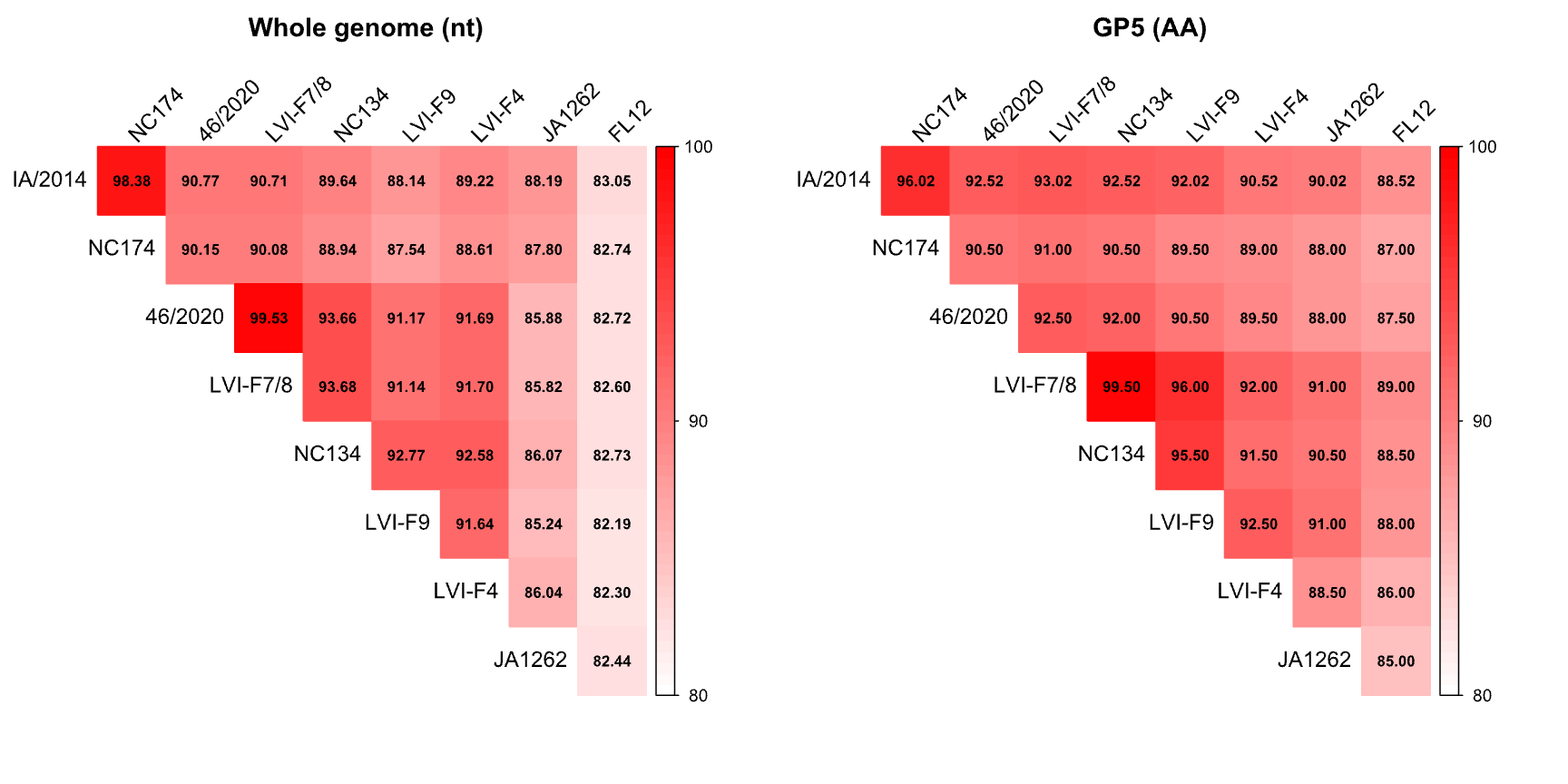


**Supplementary Figure 1.** Pairwise sequence identity among PRRSV-2 isolates. Heatmaps show pairwise nucleotide identity across complete genomes (left) and amino acid identity of the GP5 protein (right) for all inoculum-derived viral sequences. The color scale ranges from 80% to 100%, with higher identity indicated by darker red shading, illustrating variation from closely related to more divergent isolates.


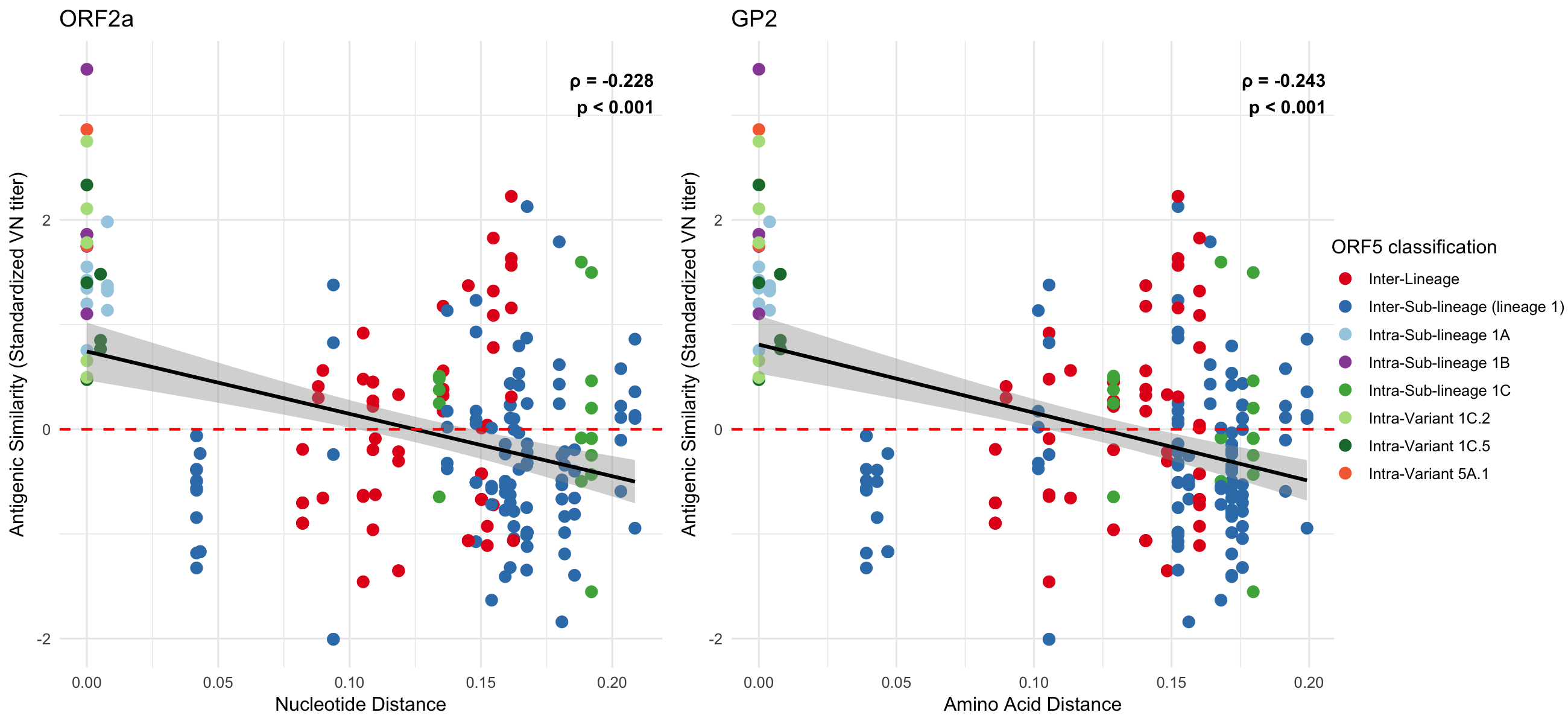


**Supplementary Figure 2.** Correlation between standardized VN titers and genetic distances (ORF2a nucleotides, GP2 amino acids). Red dashed line: mean VN titer. Black lines: linear regression with 95% CI. Upper-right: Spearman’s ρ and p-value. Point colors indicate ORF5-based variant-to-lineage classifications.


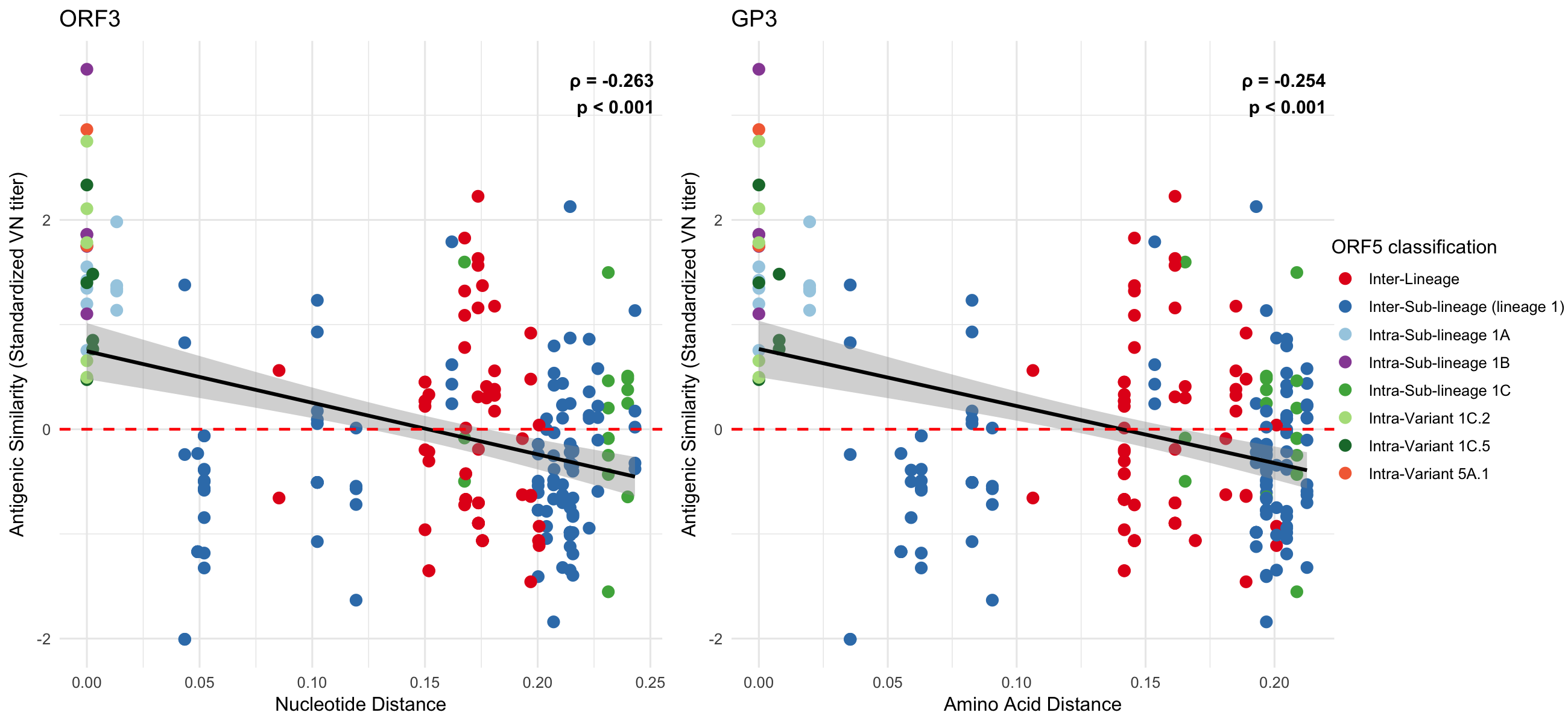


**Supplementary Figure 3.** Correlation between standardized VN titers and genetic distances (ORF3 nucleotides, GP3 amino acids). Red dashed line: mean VN titer. Black lines: linear regression with 95% CI. Upper-right: Spearman’s ρ and p-value. Point colors indicate ORF5-based variant-to-lineage classifications.


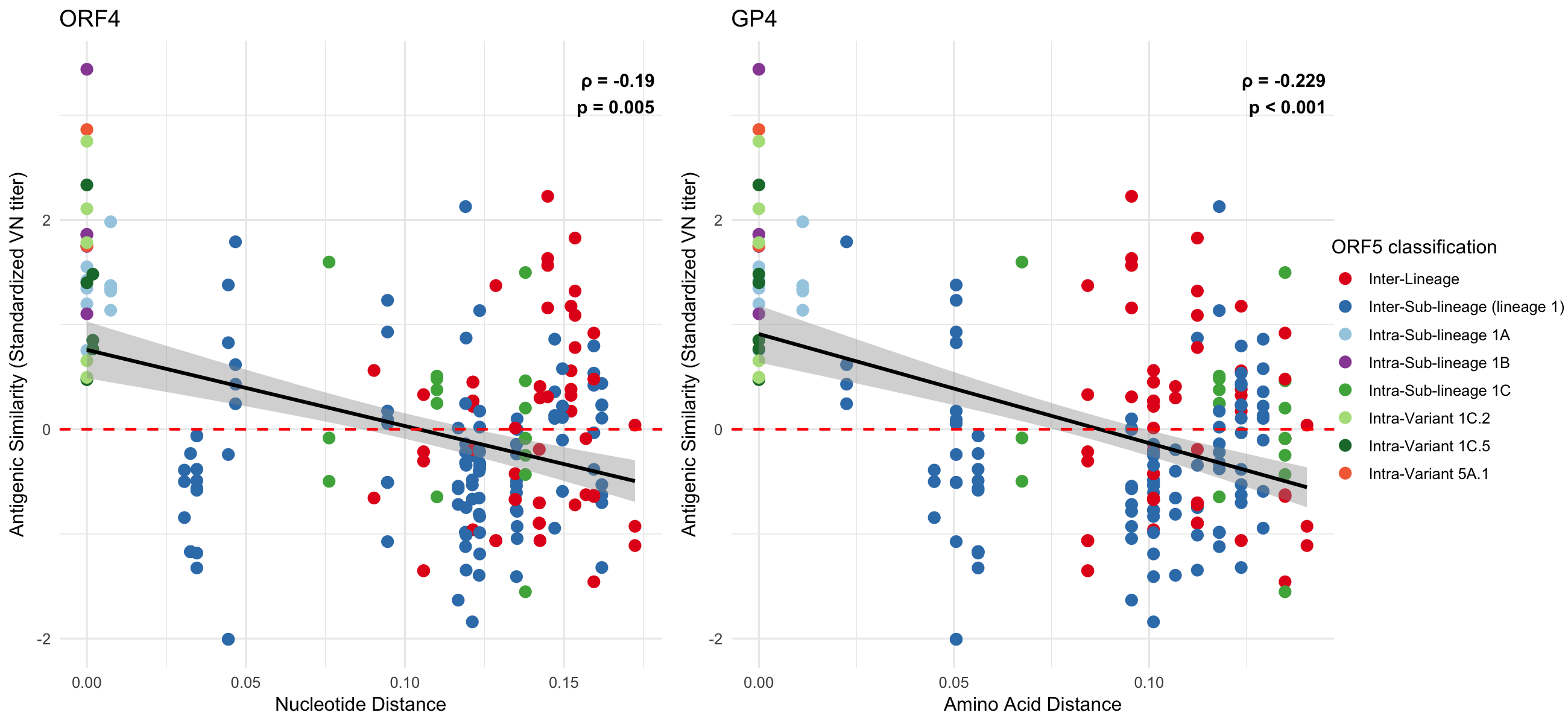


**Supplementary Figure 4.** Correlation between standardized VN titers and genetic distances (ORF4 nucleotides, GP4 amino acids). Red dashed line: mean VN titer. Black lines: linear regression with 95% CI. Upper-right: Spearman’s ρ and p-value. Point colors indicate ORF5-based variant-to-lineage classifications.


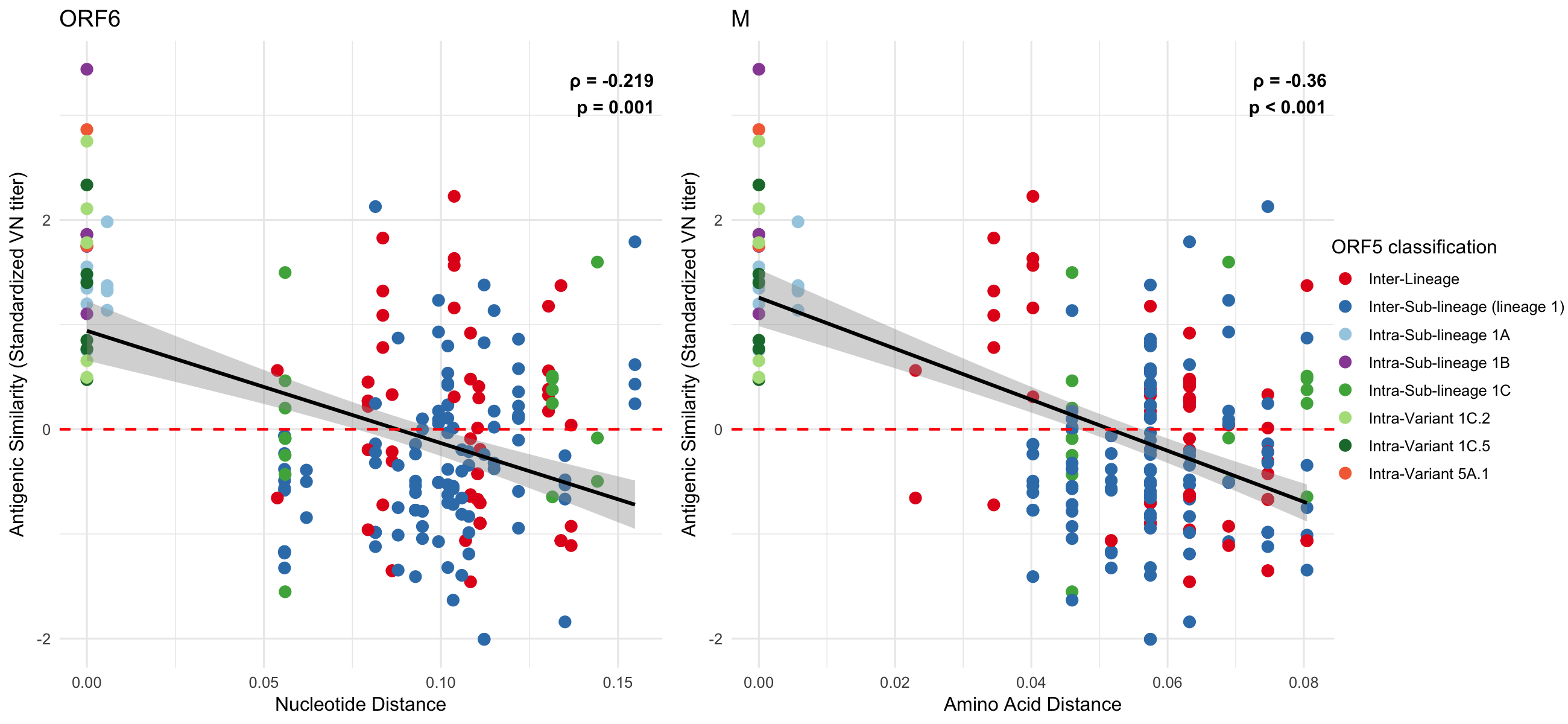


**Supplementary Figure 5.** Correlation between standardized VN titers and genetic distances (ORF6 nucleotides, M protein amino acids). Red dashed line: mean VN titer. Black lines: linear regression with 95% CI. Upper-right: Spearman’s ρ and p-value. Point colors indicate ORF5-based variant-to-lineage classifications.


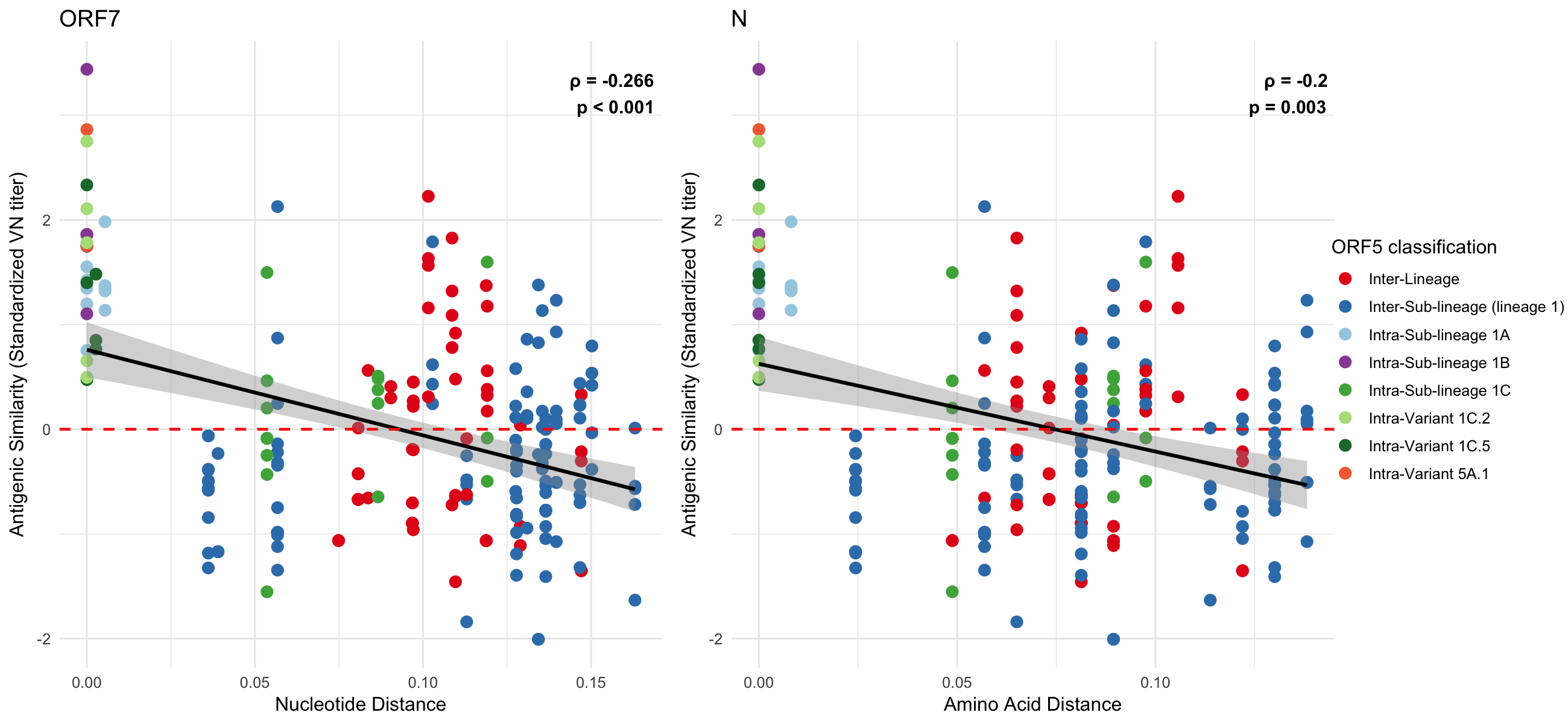


**Supplementary Figure 6.** Correlation between standardized VN titers and genetic distances (ORF7 nucleotides, N protein amino acids). Red dashed line: mean VN titer. Black lines: linear regression with 95% CI. Upper-right: Spearman’s ρ and p-value. Point colors indicate ORF5-based variant-to-lineage classifications.


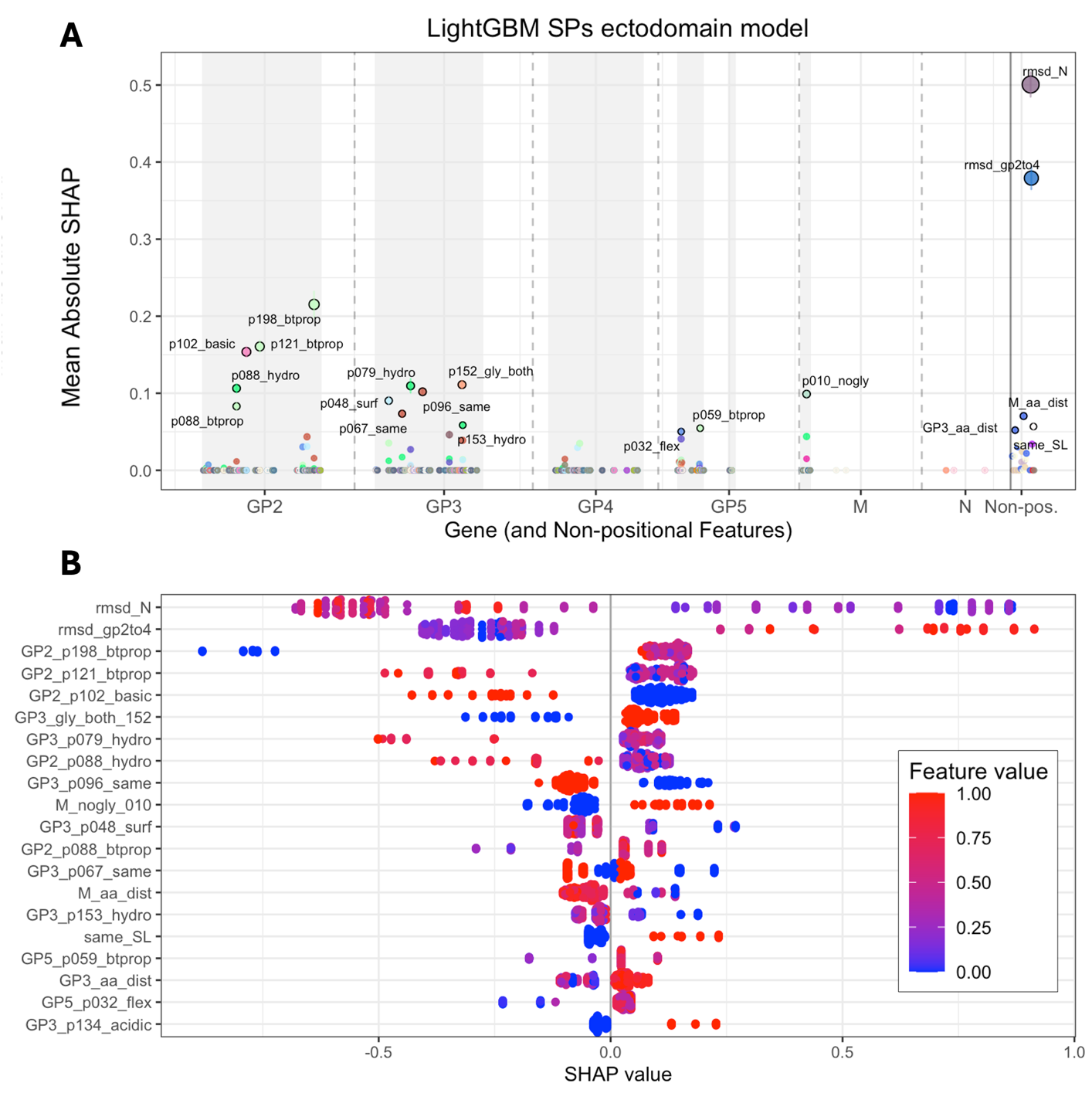


**Supplementary Figure 7.** Key features of the top structural protein ectodomain LightGBM model with features from RMSD-based structural comparisons. (A) Residue-wise feature importance (SHAP) across GP2–N; grey regions = ectodomains, right of dashed line = global features. (B) SHAP beeswarm plots showing feature contributions to predicted VN titers.


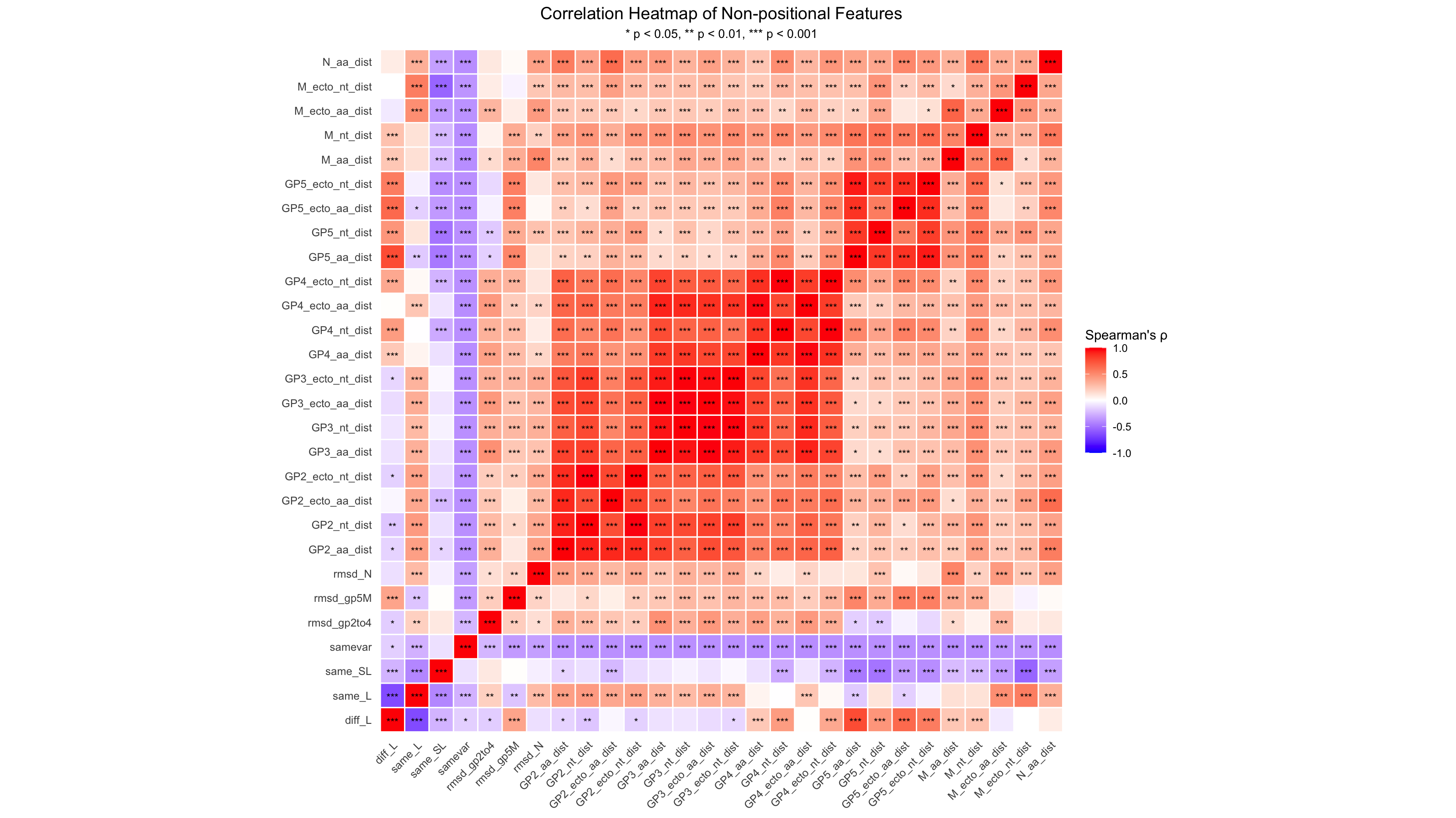
 **Supplementary Figure 8.** Heatmap showing Spearman correlations (ρ) among all non-residue features. Colors indicate correlation strength, and asterisks denote significance levels.
